# Cost effectiveness of a computer-delivered intervention to improve HIV medication adherence

**DOI:** 10.1186/1472-6947-13-29

**Published:** 2013-02-28

**Authors:** Raymond L Ownby, Drenna Waldrop-Valverde, Robin J Jacobs, Amarilis Acevedo, Joshua Caballero

**Affiliations:** 1 Department of Psychiatry and Behavioral Medicine, Room 1477, Nova Southeastern University, Fort Lauderdale, FL 33316, USA; 2 Nell Hodgson Woodruff School of Nursing, Emory University, Atlanta, GA, USA; 3 Center for Psychological Studies, Nova Southeastern University, Fort Lauderdale, FL, USA; 4 College of Pharmacy, Nova Southeastern University, Fort Lauderdale, FL, USA

**Keywords:** HIV, Cost effectiveness analysis, QALY, Computer intervention, Medication adherence

## Abstract

**Background:**

High levels of adherence to medications for HIV infection are essential for optimal clinical outcomes and to reduce viral transmission, but many patients do not achieve required levels. Clinician-delivered interventions can improve patients’ adherence, but usually require substantial effort by trained individuals and may not be widely available. Computer-delivered interventions can address this problem by reducing required staff time for delivery and by making the interventions widely available via the Internet. We previously developed a computer-delivered intervention designed to improve patients’ level of health literacy as a strategy to improve their HIV medication adherence. The intervention was shown to increase patients’ adherence, but it was not clear that the benefits resulting from the increase in adherence could justify the costs of developing and deploying the intervention. The purpose of this study was to evaluate the relation of development and deployment costs to the effectiveness of the intervention.

**Methods:**

Costs of intervention development were drawn from accounting reports for the grant under which its development was supported, adjusted for costs primarily resulting from the project’s research purpose. Effectiveness of the intervention was drawn from results of the parent study. The relation of the intervention’s effects to changes in health status, expressed as utilities, was also evaluated in order to assess the net cost of the intervention in terms of quality adjusted life years (QALYs). Sensitivity analyses evaluated ranges of possible intervention effectiveness and durations of its effects, and costs were evaluated over several deployment scenarios.

**Results:**

The intervention’s cost effectiveness depends largely on the number of persons using it and the duration of its effectiveness. Even with modest effects for a small number of patients the intervention was associated with net cost savings in some scenarios and for durations greater than three months and longer it was usually associated with a favorable cost per QALY. For intermediate and larger assumed effects and longer durations of intervention effectiveness, the intervention was associated with net cost savings.

**Conclusions:**

Computer-delivered adherence interventions may be a cost-effective strategy to improve adherence in persons treated for HIV.

**Trial registration:**

Clinicaltrials.gov identifier NCT01304186.

## Background

Although advances in combination antiretroviral therapy (cART) have had a huge impact on the effectiveness of treatment for HIV infection, treatment regimens continue to require high levels of adherence. Studies of medication adherence in persons treated for HIV infection, however, show that many affected individuals do not achieve the levels of adherence needed for optimal treatment outcomes [1,2]. Social psychological theories of health behavior have been used to develop interventions to promote adherence, and many have been successful. Theories such as Information-Motivation-Behavioral Skills model, [3] the Health Belief Model, [4] and the Theory of Planned Behavior [5] as well as empirical research on factors associated with poor adherence have been the basis for various effective interventions [6,7]. These interventions have often targeted individuals’ beliefs or knowledge about the disease or its treatment, but have also targeted factors that interfere with adherence, such as depression [8].

Although effective interventions exist, they are not widely available. Even brief clinician-delivered interventions may be beyond the reach of many patients. Interventions for adherence are clearly a part of standard care for HIV infection [9] and it is likely that standard interventions may have a positive effect on patients’ adherence [10]. It is not clear, however, whether adherence interventions are routinely provided in regular clinical care. It is likely that a great deal of clinician-delivered adherence counseling is delivered in the context of hurried clinical visits during which other medical concerns must also be addressed [11]. This strategy may be less than optimal, especially in light of research that has shown that patients may remember as little as little as 50% of orally-presented information [12,13] and that memory for information provided by clinicians is related to age, education, and gender [14]. Few clinicians are likely to have the time to spend one hour providing individually-tailored information or to have the therapeutic skills to address common concerns such as substance abuse or depression as in the intervention used in this study [15] (see Additional file 1).

Computer-delivered interventions, although expensive to develop, may be delivered at low cost on existing computers and over the Internet on mobile devices. A number of electronic interventions have been shown to be efficacious in improving medication adherence [16], and one recent trial showed that a computer-delivered intervention provided in a clinic was effective in improving medication adherence in patients treated for HIV [17].

Electronically-delivered interventions have a number of potential advantages over clinician-delivered interventions. The ongoing cost of maintaining an application on local computers or a server can be low, and computer-based interventions may be able to implement substantially similar interventions with much smaller investments of clinician time. By doing so, busy clinicians may be released from routine educational duties to cope with more complex problems that demand their attention. It is possible that if such an intervention were routinely available clinicians might spend less time in adherence counseling with patients. A computer-delivered intervention would not replace clinician efforts, but reduce the demands on their time made by routine educational tasks and allow them to provide critically important interventions to patients. Further, existing data on computer-based information resources have shown that improved access to information may have a positive effect on patient knowledge and ability to interact with physicians [18]. Electronically-delivered interventions provided on the Internet can be available in real time to patients as they are needed or as patients have the opportunity to consult them.

It could thus support clinicians’ work with these patients and even empower patients to work more actively with clinicians. Intensive interventions requiring multiple sessions and substantial clinician time, for example, may cost as much as $4,000 per year per patient [19]. Even with the costs of clinician-delivered adherence interventions, the interventions may actually reduce total net costs [19] but in spite of cost savings adherence interventions may not be widely deployed because of lack of trained personnel. Further, adherence interventions may be difficult to deliver to persons in rural areas due to the need to travel long distances in order to receive treatment [20].

We developed a computer-delivered adherence intervention that focused on improving participants’ HIV-related health literacy as a strategy to increase their medication adherence. The hour-long intervention was delivered on stock touch screen computers (Hewlett-Packard TouchSmart series; Palo Alto, California) and only required that participants interact with it by touching large buttons on the computer screen. In order to keep development costs to a minimum, the intervention utilized low-cost or free media and was developed in off-the-shelf software used for computer training and simulation (Captivate®, Adobe Corporation, San Jose, California).

Content for the intervention was first developed by reviewing popular patient education materials available commercially, online, and from advocacy organizations. The materials were then reviewed by a multidisciplinary team that included physicians, nurses, psychologists, a pharmacist, and a social worker. Resulting content was organized into concept-based sections that focused on basic information about viral replication and transmission, on mechanisms of drug action, use and interpretation of laboratory values, the meaning of 95% adherence, factors related to motivation (such as coping with depression or substance abuse), and strategies for maintaining adherence. Participants in the study completed the intervention in a single session that required approximately one hour.

Consistent with cognitive load theory [21-23] and principles of multimedia education [24], material was presented in small segments (e.g., one portion of the viral life cycle) and followed by assessing participant understanding through multiple choice questions. To enhance learner engagement and learning and reduce demands on literacy skills, material was presented in short passages of text supported by pictures, illustrations, and an animation supplemented with narration played on the computer’s speakers. When a participant failed to answer an assessment question correctly, the material was immediately retaught after displaying a personalized message employing the participant’s first name and a statement such as “That’s not quite it. Let’s go over that again.” All material with the exception of technical terms such as “protease inhibitor” was presented at a sixth grade reading level (Flesch-Kincaid readability formula as implemented in Microsoft Word®). Interactions with the computer only required that participants tap on the computer screen, thus keeping computer skills required to a minimum.

The computer-based intervention was first developed via expert consensus on content and format and then tested for its usability and acceptability with several groups of potential users. After several rounds of assessment and revision, the intervention was judged acceptable by patients and was used in the study. Participants completed the intervention in a single one-hour session. It was intended for use by patients at any point in their treatment although it might be most useful for patients beginning treatment. Although it was developed as an intervention to be reviewed once, in future research we will evaluate how long its effects on intervention persist and will consider development of a booster or review intervention designed to target maintenance of high levels of adherence.

More detailed information on the intervention and its effectiveness is available in a paper [15] and a presentation with illustrations of the intervention computer set up and example screens from the intervention itself is available online [25]. A supplement to this paper includes illustrations of screens viewed by participants in the study of the intervention Additional file 1.

The intervention’s effects on adherence were assessed using an electronic pill bottle that automatically recorded the date and time of each opening (Medication Event Monitoring System, or MEMS; Aardex, Ltd, Sion, Switzerland), providing an evaluation of adherence for the month before and the month after participants completed the intervention. Although the MEMS system is not a perfect measure of adherence, previous studies have shown that the MEMS index is closely related to viral load [26]. Participants’ demographic information (age, education, race, gender), psychosocial status on such variables as social support and depression, and cognitive functioning were assessed at the time of study enrollment, allowing us to take these variables into account in understanding participants’ response to the intervention. Results based on 118 participants who completed the intervention and follow-up visits (of a total of 124 who entered the study and 120 who completed the intervention) showed that participants with less than 85% adherence (mean model-adjusted baseline adherence was 58%) at baseline improved their adherence an average of 10% [15]. While a modest absolute change in behavior, the observed change represented a medium effect size consistent with other clinician-delivered interventions to improve adherence [27] and was similar in magnitude to that observed in a study of another computer-delivered intervention for older persons with memory impairments [28]. Given the possibility that computer-delivered interventions may have effects similar to those of interventions that make greater demands on clinician time, may cost more to develop, but be more readily deployed to a large audience, a determination of the cost-effectiveness of the computer-delivered intervention was judged important. The purpose of this study was to assess the cost effectiveness of a computer-delivered intervention targeting health literacy and adherence in persons treated for HIV infection.

## Method

### Overview

**Figure 1:**
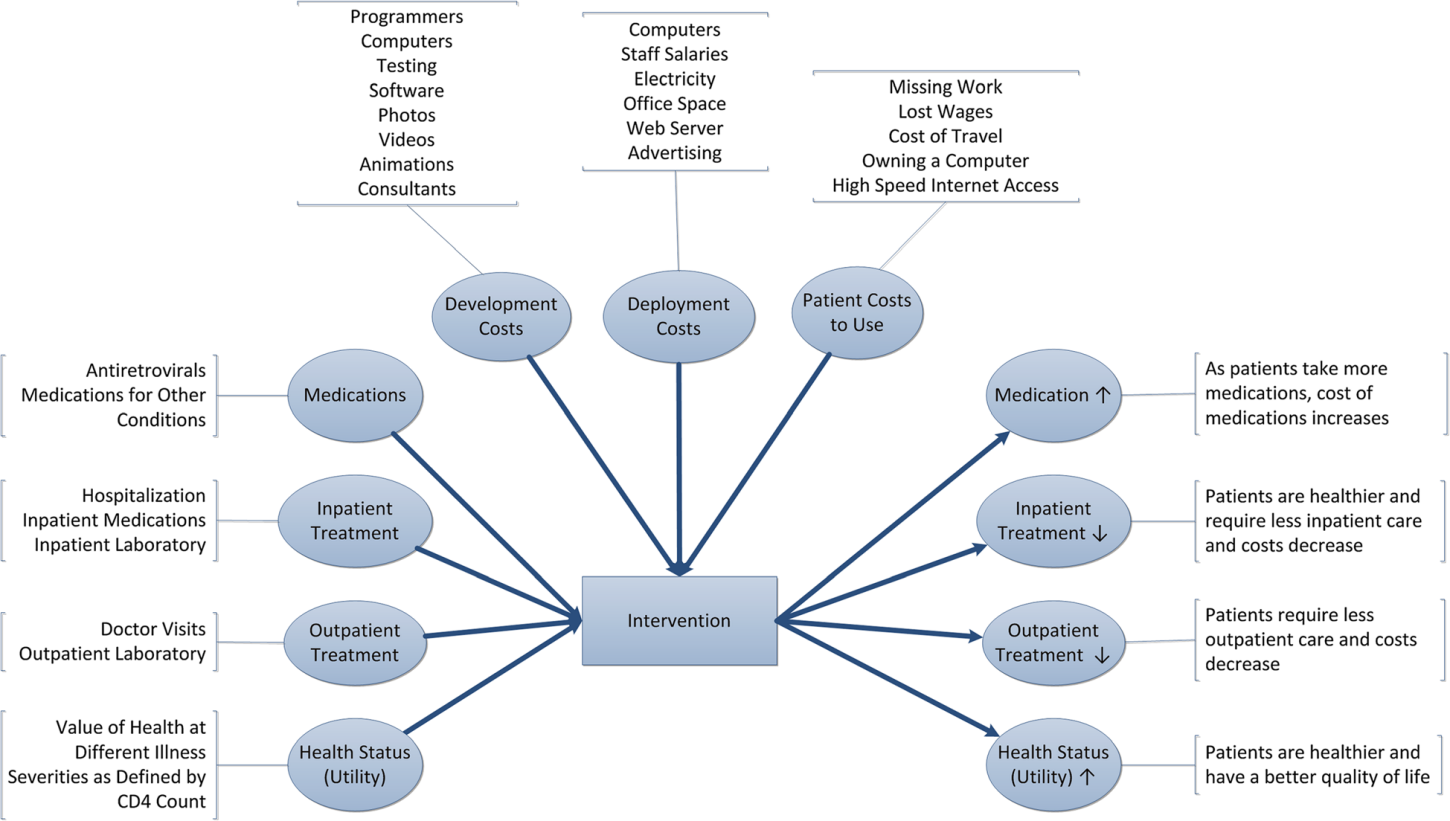
Overview of cost effectiveness analysis.

In this study, the costs of developing the intervention were known and adjusted from the actual costs of the research grant to take into account purely research-related expenses. Costs of deploying the intervention were calculated in two formats (as an information kiosk in a clinic or office and as a web-based application available on computers or mobile devices) and under two possible levels of utilization. All analyses were completed from the societal perspective (taking into account all total economic costs and benefits without regard for payer). Figure 1 provides an overview of factors related to costs involved in treating patients with HIV and their quality of life as health utilities (left portion of figure), the costs of developing and deploying the computer-based intervention (top portion of figure) and the effects of the intervention on treatment costs and quality of life as health utilities (right portion of figure). A list of the sources of the estimates used in analyses is provided in Table 1.

**Table 1:** Sources of estimates used in analysis

**Estimate**	**Source**	**Effect of intervention**
***Treatment Costs***		
Cost of Medications	Based on participants’ actual antiretroviral regimens with costs based on current average prices in 2012[39]. The average annual cost per patient if fully adherent was $22,675.	Increased use of medications because of increased adherence increases costs of medications
Inpatient and Outpatient Treatment by CD4 count	Based on data from Gebo et al. [30] adjusted to 2012 dollars reduced by medication costs that were calculated directly (above). Costs vary by patient CD4 count, with an overall average annual cost of $13,296	Improved health may increase cheaper outpatient care use but decrease more expensive inpatient care
***Development Costs***		
Salary, Wages, Software, Consultants, Hardware, Space, Media, Testing, Test Deployment for study	Actual costs as recorded in records of grant expenditures (detailed in Table 3)	
Costs to participants during development	Loss of salary and wages – estimate of lost salary and wages by participants who reported working based on their education level [32].	
	Cost of transportation -- from actual participant reimbursement during development ($5.00 for daily bus fare in our area).	
***Deployment costs – Office Kiosk Scenario***		
Space, computers, maintenance, staff, electricity	Cost of office space from based on industry report [36]	
	Cost of computer purchase, operation, and maintenance based on industry report [35]	
	Staff time costs based on average hourly wage for medical assistant plus fringe benefits and administrative costs (from US Bureau of Labor Statistics and institutional fringe benefit and administrative cost rates)	
Software License	Nominal fee assumed to defray costs of duplication of media and shipping (assumption)	
Transportation for rural patients	Average distance for rural patients to visit specialist practitioner from Rosenthal et al. [37] and average cost per mile of transportation [38] via mid-size sedan per the American Automobile Association	
Lost salary and wages	Based on estimates obtained during development for representative number of patients based on time lost and salary calculated from participants’ educational status [32]	
Cost of computer and high speed Internet access at home	Considered to be part of patients’ regular cost of living, as the actual cost of one hour of computer and Internet usage is small	
***Deployment costs – Web Deployment Scenario***		
Server operation and maintenance	Page et al. 2012 [44].	
Advertising	Page et al. 2012 [44]; amount increased for larger number of users	
Technical Support	Page et al. 2012 [44].	
***Utilities***		
Numeric value of a specific health status (more or less ill) based on immune function reflected in CD4 (immune cell) count	Kauf et al. 2008 [29].	Improved adherence will improve immune function and thus health status, enhance patients’ quality of life

**Figure 2:**
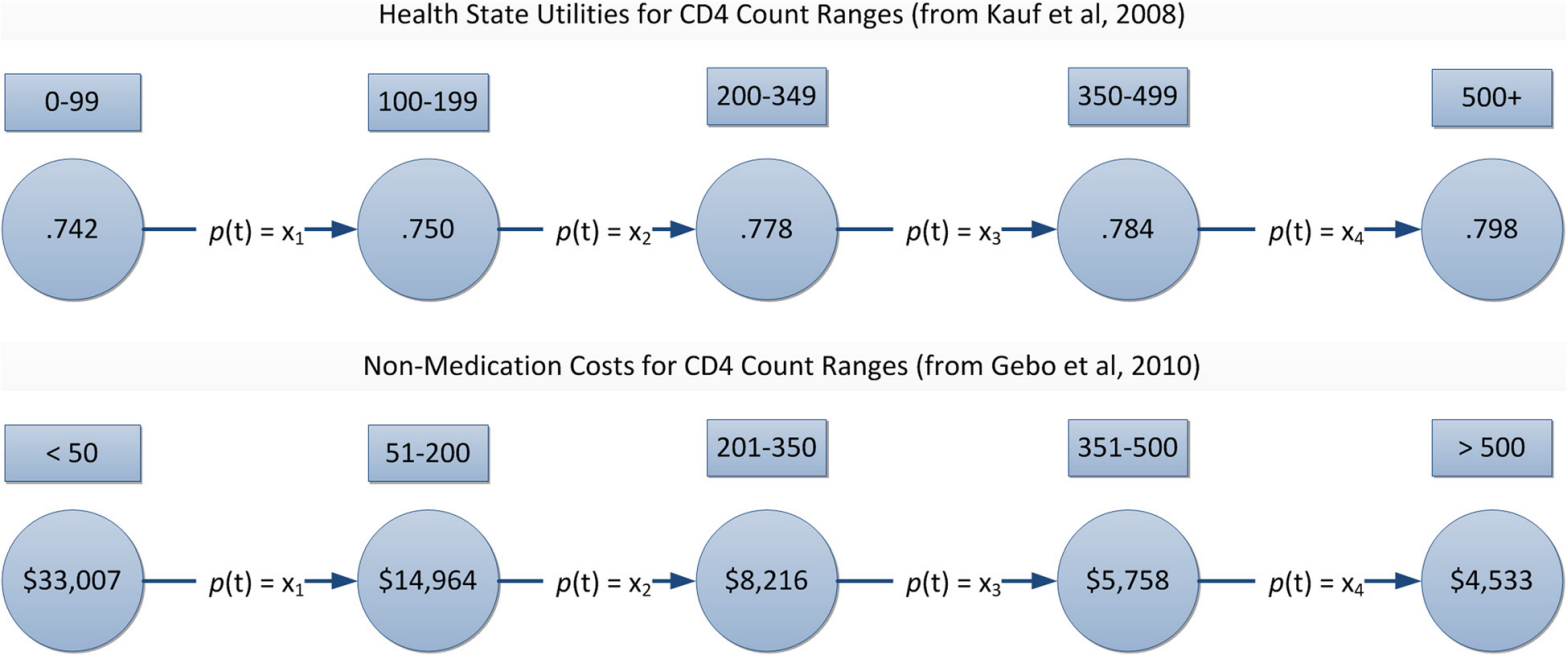
**Utilities and costs for health states defined by CD4 counts.***Note:* Each expression labeled “*p*(t)” corresponds to a probability drawn from the effectiveness scenarios listed in Table 2. For example, element *p*(t) for x_1_ (the probability that the intervention would be associated with an increase in CD4 causing movement of patients from one group to another) would be 5% for the minimally effective scenario (first line of Table 2).

Calculation of the effectiveness of the intervention was limited by lack of precise data on how changes in medication adherence might affect participants’ health costs or status. Data on healthcare-related costs could not be drawn from the parent study of the intervention as the scope of the original project did not allow this information to be collected, and data on adherence-related health status could not be drawn from the parent study due to its short duration. Data are available from other sources, however, on costs and health state utilities associated with CD4 count ranges [29,30] and the effects of adherence on viral load and CD4 counts [31]. These were used to calculate a Markov model of changes in health costs and utilities resulting from increases in adherence resulting from patients’ exposure to the intervention (see Figure 2). As estimates of CD4 count changes in response to the intervention vary widely and were not available in our data, a range of possible effects of the intervention were evaluated with respect to changes in costs and utilities (Table 2).

**Table 2:** Probability change scenarios

	**Probability of change in disease state from lower CD4 range to next higher (see Figure**2**)**				
Effectiveness of Intervention	*p*(t) x_1_	*p*(t) x_2_	*p*(t) x_3_	*p*(t) x_4_	Average
Minimally	5%	0%	0%	0%	1%
Slightly	5%	1%	0%	0%	2%
Moderately	10%	5%	5%	0%	5%
Highly	20%	10%	5%	0%	9%

*Note:* Each *p* represents an assumed probability that the intervention would increase a patient’s CD4 count sufficiently that they would move to the next higher CD4 group (see Figure 2). The expression *t* in parentheses indicates that the probability refers to a transition, while each *x* with a subscript indicates which of the four possible transitions is represented. For the minimally effective scenario, the probability represented as “*p*(t) x_1_” thus indicates that there is a 5% chance that a person in the lower group might move to the next higher group. The average column is the mean of the probabilities for each possible change in CD4 and was used for estimation of changes in costs and utilities.

In a cost effectiveness analysis, total costs of the intervention (development and deployment costs adjusted for changes in health care expenses) were evaluated for the two deployment formats with two levels of utilization. In a cost-utility analysis, likely change in health state utilities due to increased adherence were estimated and used to calculate the cost of the intervention per quality-adjusted life year (QALY).

### Costs

Costs were calculated in two ways in order to allow evaluation of a range of possibilities. As the project was supported by a grant, a precise account of all expenditures in relation to the development and testing of the intervention was available. Costs of staff salary, payments to participants, and administrative overhead are listed in Table 3. Costs were calculated as a portion of the total project costs, as the total expenditures under the grant were larger than those that would have otherwise been required due to its research purpose. Some costs in this second estimate are reduced to reflect more accurately development costs in a non-research setting. Costs therefore were reduced by the amounts of participant payments for baseline and follow-up visits as these might not have occurred in a commercial development setting and the amounts budgeted for presentation of research results at professional meetings and compensation paid to a research consultant.

**Table 3:** **Adjusted development costs**^**a**^

	
Salaries	$152,323
Fringe	$39,909
Total Salaries	$192,232
Participant Payments	$6,584
Participants’ Lost Wages	$742
Supplies, Equipment, Computers	$4,647
Software	$500
Total Direct Costs	$204,704
Administrative Support Costs @ 44.8% of Total Costs	$76893
Total Development Costs	$296,411

^a^Total costs of project reduced for costs specific to research, including costs of research consultant and participant visits only related to research.

### Lost salary and wages

Development costs were increased to reflect participants’ lost salary and wages resulting from time taken off from work to participate in the visit during which they completed the intervention. This estimate of total development costs was used in subsequent calculations.

Economic costs of lost wages to participants were calculated based on the mean annual wage for each participant’s level of education [32] for those who reported working outside the home for pay. Twenty-two participants reported working for pay outside the home, and lost wages were calculated for two hours (one hour for the intervention and one hour average time for the round trip from work or residence to the study site). Total lost wages for the study was $742. This amount was included in estimates of the total cost of developing the intervention. Participants were compensated for their effort in developing the intervention at the rate of $50 per visit. The cost of paying participants for the study visit during which they completed the intervention is also included as part of total development costs.

### Administrative costs

The administrative costs charged to the grant were based on the negotiated rate between our university and the US government and included to represent the administrative overhead involved in the development process. These reflect the costs of providing office space, utilities, and administrative support for the development project.

### Deployment costs

An important factor in understanding the cost-effectiveness of computer-delivered interventions is that while initial development costs may be large compared to clinician-delivered interventions, their subsequent deployment can readily make them widely available at low cost. The costs of deployment of the intervention for routine use in clinical practice, both in the form it was developed (information kiosk style application on a touch screen computer) and on the Internet (on a server with the application accessible to a wider audience, including advertising costs) were therefore estimated.

For the office-based information kiosk scenario, costs were estimated based on two scenarios of the frequency of intervention use. One estimate (low use office) was based on estimates of the percentage of new patient ambulatory care visits (assuming all patients were treated for HIV and all would receive the intervention). A second estimate (high office) was based on percentage of all ambulatory care visits that involve health education. Based on an average number of patients per seen day of approximately 30 [33] and on data showing that 15% of office visits are for new patients [34], under the first utilization scenario 2 patients seen per day in a clinical office might be eligible for the computer-based intervention. Conversely, data show that 40.3% of ambulatory care visits include provision of health education [34], from this perspective suggesting that as many as 12 patients per day (of 30 seen) might be eligible for the computer intervention. The number of visits eligible for the computer-based intervention under both scenarios for the period of time evaluated was used to calculate the total annual cost of deploying the intervention. Under the low use scenario, it was judged that one computer kiosk would be sufficient, while under the high use scenario it was estimated that at least three computer kiosks would be required to accommodate the hypothesized level of utilization.

Costs of deploying the intervention in a kiosk in a clinician’s office include the expense of computer purchase and maintenance, estimated in an industry report as $2,200 per year [35]. Other costs include a nominal licensing fee charged by the software supplier to defray ongoing costs of software distribution, costs of office space estimated for a 8 foot by 8 foot office (64 square feet) using a cost of $23.11 per square feet (including utilities and maintenance based on average office costs for South Florida in the United States reported in an industry publication [36]). Staff time required to demonstrate the intervention and monitor patients under the low use scenario was estimated to be one hour per day at the national annual average medical assistant salary of $14.61 per hour obtained from the web site of the US Bureau of Labor Statistics, increased by 26.2% for fringe benefits and an additional 44.8% for administrative overhead. For the scenario under which 40% of office visits involved patient education, it was assumed that for the larger number of patients a larger number of computers and offices would be needed (three compared to the one used for the alternative scenario). Computer and office-related costs were therefore increased by a factor of three in calculating costs in this scenario. Since it might be necessary for rural users to travel to practitioners’ offices to use the intervention when it is deployed as an information kiosk, transportation costs were calculated based on the average distance that rural patients travel to reach a specialist physician [37] and the average cost per mile of automobile travel in the US (59.6 cents) [38]. Transportation costs were included for a supplementary scenario in which all users were in rural areas and were required to travel by automobile to a clinician’s office to use the intervention. (These analyes are included in supplementary tables included in Additional file 2.)

### Costs associated with medications and clinical care

While generally desirable from a clinical and public health point of view, an increase in patient adherence is likely to be associated with greater medication costs since patients will take their medications more regularly and consume more medications. In order to evaluate the impact of the computer-based intervention on total treatment costs, each participant’s monthly medication costs was calculated using data provided by participants on their medications and using monthly average wholesale medication costs for 2012 in estimates provided in a recent treatment guideline [39]. Total average monthly medication costs were calculated as the total of costs for each participant assuming 70% adherence (the level of adherence commonly found in studies of adherence in this population [7]) divided by the number of participants, with the increase related to the intervention calculated as the 10% effect of intervention-related increase in medication consumption for the proportion of patients whose medication adherence increased.

Costs related to care were based on previously established costs for all health care costs except antiretroviral medications (inpatient, outpatient, laboratory, and non-HIV-related medications) for ranges of CD4 cell counts [30] adjusted to 2012 dollars. Changes in costs related to changes in adherence were calculated based on previously reported values derived from a computer model of the relations among adherence, viral load, and CD4 count [31]. As our sample size did not provide a reliable way of establishing the effect of the intervention of persons with lower ranges of CD4 counts sensitivity analyses were used to evaluate a wide range of possible changes in CD4 counts in response to the intervention.

### Costs and utilities related to change in adherence

Non-medication related costs related to health status were calculated under four possible scenarios of change in health status based on CD4 count (less than 50 cells/mm^3^, 100–199, 200–349, 350–499, and greater than 500; see bottom half of Figure 2). For example, in the “minimally effective” scenario (see Table 2) which hypothesized the smallest intervention effect, it was assumed that 5% of persons with CD4 counts less than 50 would show a treatment effect resulting in their moving from the less than 50 group to the group with CD4 cells in the range of 51 to 200 (this 5% transition probability is represented in Figure 2 as *p*(t)x_1_, ie, the transition probability for the first possible change from one group to another). Based on costs reported by Gebo et al. [30], the per person decrease in annual medical costs (excluding medications, but including inpatient, outpatient, and laboratory costs) for the transition between these two groups would be $18,043 (the difference between $33,007 for the first state, and $14,964 for the new state; see lower portion of Figure 2). Change in costs associated with the intervention’s effect was thus calculated as 5% of this amount (again using the transition probability represented as *p*(t)x_1_); changes in per person costs for each state (when scenarios assumed transitions occurred for several groups) were averaged across CD4 groups and used in subsequent calculations.

Better health resulting from improved adherence was assumed to result in some participants being able to return to work. Issues related to return to work for persons with HIV/AIDS are complex, including such factors as loss of disability benefits including medical insurance with return to work, difficulties in finding child care, as well as enhanced self-esteem from the return to work [40,41]. Although with implementation of a return to work program a substantial number of persons with HIV/AIDS may be able to return to partial employment [42], one study showed that only about 15% of patients returned to full time work over two years [43]. In order to account for the possible economic benefits of patients returning to work, the effects of 15% of participants returning to full-time work (based on estimates of salary or wages for each person based on their educational attainment) were included in analyses.

In a similar way, changes in health utilities were also based on previously determined utility values [29] for states defined by CD4 count (top half of Figure 2) and the probability of a patient’s status changing in response to the intervention. For example, the change in health utility for a patient whose CD4 status changed from less than 100 to the range of 100 to 199 would be 0.008 (the difference in utility between the first and second states). As with calculation of costs, the net change for health utility was calculated as the product of the change and the probability (*p*(t)x_i_) that patients would change their status. Values were calculated for each possible transition to a different health status, and an average of amounts was used for calculating aggregate values. The transition probabilities used in each of these effectiveness-related scenarios are presented in Table 2. In analyses it was assumed that the change modeled by the transition probability would affect the given number of participants for the period during which the intervention was assumed to have an effect. In the analyses presented here, this period was assumed to be six months. In supplementary analyses evaluating the effects of different durations of intervention effectiveness, the period varied from one to 12 months. In all analyses, it was assumed that the only effect of the intervention was to increase the probability that a patient would make the transition from a lower to a higher CD4 and that persons with high CD4 levels probably also had high levels of adherence and thus would show little or no response to the intervention, as shown in the original study [15]. Although it is likely that in a clinical setting some patients might make a transition from higher to lower group, this possibility was not explicitly modeled.

The format of the intervention allows it to be made available to potential users in several ways. As developed, the intervention was presented to users on a stationary computer; the intent was to allow for the intervention to be implemented in a clinician’s office as an information kiosk, with patients completing the intervention in the physician’s office as part of routine care. The intervention can, however, be deployed on a web site that would allow much wider access by large number of patients. Another group of investigators has presented cost data for a web-based adherence intervention [44], and we used their estimates in calculating the cost of deploying the computer-based health literacy application so that it was possible to compare deployment costs for several options. Costs of the web-based deployment were calculated for the same number of users as employed in cost calculations for the clinical office scenario as well as for a much larger number of users as might be expected for a web-based application. For this larger number of users, costs were held constant except for advertising costs. Advertising costs were increased by ten times (based on the assumption that ten time increase in advertising would be required to reach the necessary number of potential participants) to reflect the need for greater publicity in order to ensure that a substantially larger number of participants would use the web-based intervention. The costs of deploying the intervention on the Internet were calculated using the same estimates of staff, server hosting advertising, and technical support provided by Page et al. [44] for a web-based adherence intervention. Analyses of annual costs at two levels of utilization were calculated for the web-based deployment across the same levels of effectiveness used for the office or clinic based format (Table 4).

**Table 4:** **Semiannual costs for web deployment**^**a**^

	**Low utilization (540 users)**	**High utilization (1,620 users)**
Project Coordinator	$17,160	$17,160
Server Hosting	$150	$450
Advertising	$510	$5,100
Technical Support	$10,398	$20,796
Total Costs	$28,218	$43,506
Deployment Cost per User	$52	$27

^a^Costs for low utilization scenario are drawn from Page et al. [44]. Costs for high utilization scenario are increased for server hosting, technical support, and advertising to reflect greater server use and advertising to increase number of users.

### Effectiveness

Analyses showed that the magnitude of the intervention’s effect was related to a patient’s baseline level of adherence but averaged an increase of 10% [45] in patients with baseline adherence less than 85%, similar to the increase obtained in a study of a similar intervention among elderly patients with memory impairments [46]. Participants with low levels of adherence at baseline showed greater increases in adherence than those with higher baseline levels. Because of lack of adequate data to determine the effects of changes in medication adherence on CD4 counts and thus on health utilities, sensitivity analyses were completed for a range of possible levels of effect. Scenarios were estimated as the percentage of each group with a specific CD4 count which might increase their CD4 counts so that they moved to the next higher group under the scenarios presented in Table 2. It was assumed that a proportion of the individuals with CD4 counts in the lower groups would have these levels due to poor medication adherence and thus exposure to the intervention would be more likely to result in changes in adherence and health status. It was also assumed that individuals with higher CD4 counts were more likely to already have high levels of adherence and thus were less likely to show a change in CD4 count in response to the intervention.

### Utilities

Health utilities are numeric quantities that represent the value of life adjusted for health status. In their calculation, it is assumed that one year of life with lower health is worth less to a patient than a year in perfect health. The value of a year of life whose quality is reduced by poor health might be represented by a value less than 1.00 [47]. A previous study had developed health utilities in those with HIV infection related to ranges of CD4 counts [29]. In evaluating health utilities it was assumed that an increase in medication adherence would result in improved health as indicated by a higher CD4 cell count. A net change in utilities for each CD4-defined group was calculated as the product of the probability of a change in a patient’s life quality utility and the probability that their CD4 status would change in response to the intervention. Changes for each category were averaged to provide an overall net change in utility for the intervention. As completed for cost estimates related to changes in CD4 count, sensitivity analyses were completed to evaluate a range of potential effects of the intervention on adherence. QALYs were calculated based on changes in utilities as described above, making the assumption that the effect of the intervention persisted for six months. The widely-used value of $50,000 per QALY was used to judge the intervention’s cost effectiveness, although we note that values ranging up to $100,000 per QALY have been used in other studies and that other authorities have argued that the actual cost per QALY may be higher when it is evaluated in the context of the cost of other interventions commonly paid for by third party payers [48,49].

### Sensitivity analyses

In addition to the sensitivity analyses completed as evaluation of the range of levels of intervention effectiveness based on its effect patient health status (the four effectiveness levels described in Table 2), potential variations in development and deployment costs were also evaluated in sensitivity analyses by assessing the effect of a range of increases and decreases in development costs when development costs were not reduced by research-related amounts. These analyses are reported in supplementary tables for this paper. As the initial study of the intervention had only demonstrated its effects over one month, cost per QALY was evaluated for each deployment scenario over four possible durations of effectiveness, three, six, nine, and twelve months. These times were chosen based on follow-up studies of other adherence interventions [27,50,51] that have shown that adherence-oriented educational intervention effects may persist up to 18 months.

## Results

Adjusted development costs are presented in Table 3. Total direct costs for development were $204,704 which, combined with administrative costs yields a total project cost of $296,411. For the 124 participants, this resulted in a development cost per participant of $2,390. Assuming an average increase of 10% adherence for each participant, the development costs thus were $239 for each 1% increase in adherence.

As the effect of the intervention was assumed to persist for six months, deployment cost calculations are based on this period. Semiannual costs of deploying the intervention in a clinician’s office under two utilization scenarios are presented in Table 5. The cost of providing the intervention (exclusive of development costs) to each patient under the low and high utilization scenarios was $60, for a cost per 1% increase of $6. Costs of deploying the intervention on the Internet under two utilization scenarios are presented in Table 4. For the same number of users as in the high utilization scenario in a clinician’s office (540 users semiannually), the cost per user for Internet delivery is $52 with a cost of $5 for 1%. For a larger number of semiannual users, however, costs drop substantially even with an increase in advertising costs, to $27 per user, and a cost of $3 for each 1% increase.

**Table 5:** Semiannual costs for information kiosk deployment (office or clinic)

	**Low utilization (540 users)**	**High utilization (1,620 users)**
Computer per year (includes electrical consumption, maintenance, software)	$2,200	$6,600
Licensing	$50	$150
Office space @ $23.11 / sq ft	$8,874	$26,623
Staff support (medical assistant)	$3,181	$9,543
Loss of salary and wages from office visit	$18,202	$54,607
Total cost of deployment	$32,507	$97,522
Total users for six months	540	1620
Deployment cost per patient	$60	$60

Cost utility analyses for the information kiosk deployment strategy are presented in Table 6. Taking into account all costs and benefits, including increase in medication costs and decrease in other healthcare costs yielded a net cost of the intervention for the minimally effective scenario of $255,032 for a cost per QALY of $67,469. The cost per QALY declines with increases in intervention effectiveness and a larger number of users. The highest effectiveness level for the low utilization deployment scenario is associated with a net cost savings of $15,726, while for the high utilization scenario all costs per QALY are less than $20,000 or are negative, indicating a net cost savings.

**Table 6:** Semiannual net intervention costs and cost per QALY for kiosk deployment by efficacy scenarios

	**Low utilization (540 users)**				**High utilization (1,620 users)**			
**Cost**	**Minimally**	**Slightly**	**Moderately**	**Highly**	**Minimally**	**Slightly**	**Moderately**	**Highly**
Increase in medication costs	$7,635	$9,183	$30,611	$53,569	$22,958	$27,550	$91,833	$160,707
Decrease in IP/OP/other treatment costs	$60,895	$65,450	$152,861	$297,424	$182,685	$196,349	$458,582	$892,272
Loss of salary and wages from participation	$18,202	$18,202	$18,202	$18,202	$54,607	$54,607	$54,607	$54,607
Increase in salaries and wages resulting from return to work	$20,644	$24,722	$82,575	$144,506	$61,931	$74,317	$247,725	$433,518
Net cost of intervention (includes development and deployment)	$255,032	$247,879	$124,094	($59,443)	$172,275	$150,816	($220,540)^a^	($771,150)
Cost per user	$472	$459	$230	($110)	$106	$93	($136)	($476)
Cost per QALY	$67,469	$65,577	$32,829	($15,726)	$15,192	$13,300	($19,448)	($68,003)

^a^Numeric values in parentheses indicate negative values, i.e., net cost savings for the intervention in this scenario.

Analyses for deployment of the intervention on the Internet are presented in Table 7. While the cost per QALY is positive for scenarios with low levels of intervention utilization, higher levels of effectiveness with higher levels of utilization resulted in net cost savings. For the high utilization condition, costs per QALY at all levels of effectiveness were less than $50,000 and in several cases were associated with net cost savings (values in parentheses in Table 7).

**Table 7:** Semiannual intervention costs and cost per QALY for web deployment by efficacy scenarios

	**Low utilization (540 users)**				**High utilization (1,620 users)**			
**Cost**	**Minimally**	**Slightly**	**Moderately**	**Highly**	**Minimally**	**Slightly**	**Moderately**	**Highly**
Increase in medication costs	$15,305	$18,367	$61,222	$107,138	$45,916	$55,100	$183,655	$321,414
Decrease in IP/OP/Other treatment costs	$121,790	$130,989	$305,721	$594,848	$365,371	$392,698	$917,163	$1,784,543
Loss of salary and wages from participation	$18,202	$18,202	$18,202	$18,202	$54,607	$54,607	$54,607	$54,607
Increase in salaries and wages resulting from return to work	$20,644	$24,722	$82,575	$144,506	$61,931	$74,317	$247,725	$433,518
Net cost of intervention (including development and deployment)	$215,703	$205,526	$15,757	($289,385)^a^	$13,138	(17,393)	($586,699)	($1,502,124)
Cost per user	$399	$381	$29	($536)	$8	($11)	($362)	($927)
Cost per QALY	$57,064	$54,372	$4,169	($76,557)	$1,159	($1,534)	($51,737)	($132,462)

^a^Numeric values in parentheses indicate negative values, i.e., net cost savings for the intervention.

### Thresholds and sensitivity analyses

Thresholds for development costs that would result in a value of $50,000 per QALY were calculated for each scenario at the moderate effectiveness level. For the office kiosk low utilization scenario with 540 patients over six months, 122% of adjusted costs resulted in a value of $50,000 per QALY. For the office high utilization scenario, 267% of adjusted costs were related to the target cost per QALY. For the low utilization Internet deployment scenario, a decrease in costs to 77% of the total were associated with the target cost per QALY, while for the high utilization Internet scenario, costs inflated to 288% were related to target cost per QALY. Thus with the exception of the low utilization Internet deployment scenario, the conventional value of $50,000 per QALY was associated with increases in development costs over the unadjusted values obtained from the grant budget.

Sensitivity analyses using unadjusted costs that evaluated possible effects of a range of increases or decreases in development costs from 50% of the unadjusted value to 150% of the value costs were completed. These showed that increases in development and deployment costs affected costs per QALY so that for low levels of effect, short duration of effect cost per QALY values were very large – in excess of $1,000,000 (see Additional file 2). Cost per QALY for the 6 months’ duration used in these analyses showed that values ranged from $39,805 for the minimally effective scenario with costs at 50% of original to a $58,114 for the highly effective scenario evaluated at 150% of original costs (Additional file 2). Addition of costs of transportation (round trip travel by car to a doctor’s office in order to use the intervention) for rural patients resulted in similar values, again very large for short durations of effectiveness and cost inflations and most values lower for longer durations of effectiveness (see Additional file 2).

### Summary

**Figure 3:**
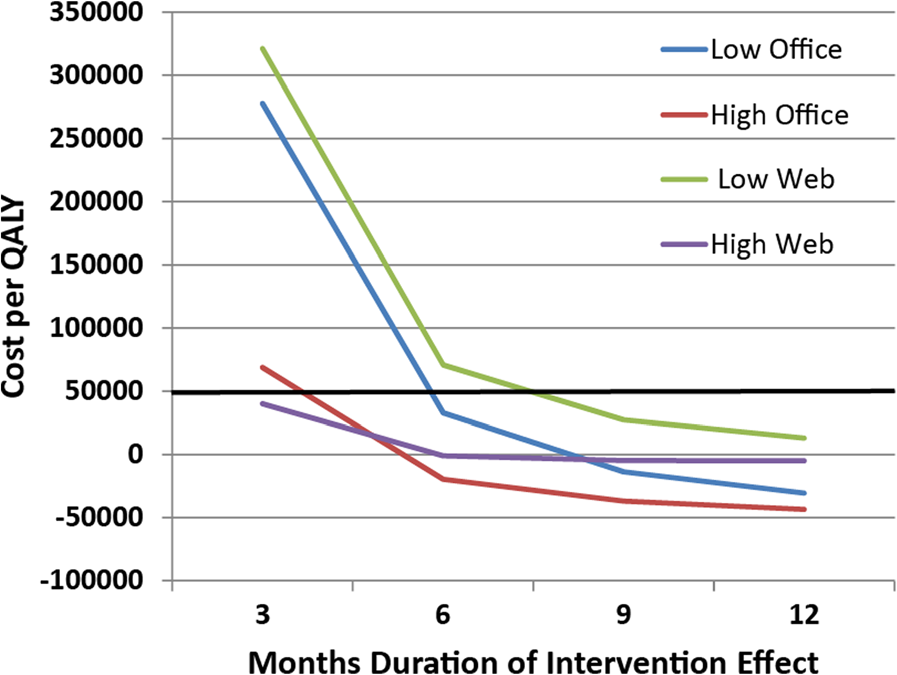
**Cost per QALY for deployment scenarios and duration of effect.***Note:* For each deployment scenario, the cost per QALY at a moderate level of effectiveness for four possible durations of interventions effect. Black line marks the $50,000 cost per QALY commonly used to assess whether an intervention is considered cost effective.

Relations among effectiveness, number of users, and duration of intervention effectiveness are illustrated in Figure 3. Cost per QALY is at a level typically considered cost effective for the high utilization office kiosk and Internet scenarios at three months and for all scenarios at six months. Longer durations of effectiveness were associated with net cost savings for both office-based scenarios and the high utilization Internet scenario. For very short durations of effectiveness such as one month, cost per QALY was very large (often greater than $1,000,000). The cost-effectiveness of the intervention thus is closely related to the time during which its effects persist, most likely due to the ongoing accrual of cost savings and improved quality of life over time. These accruals of savings over time offset the initial development costs, emphasizing the importance of the intervention’s duration of effect in understanding the relation of costs to the interventions overall cost effectiveness.

## Discussion

The purpose of this paper was to evaluate the relation of the costs of developing and deploying a computer-delivered adherence intervention to its effects in decreasing treatment costs and improving health status. A specific goal was to evaluate whether the initial high costs of development of such an intervention are offset by its benefits over time. The actual costs of developing the intervention and estimates of the costs of participants’ medications based on their actual medication regimens were used in analyses. Changes in costs related to other aspects of care (inpatient and outpatient care; laboratory; non-HIV-related medications) were estimated from another report [30], as these were not recorded for current study participants. Sensitivity analyses assessed the relation of outcomes to a range of costs, intervention effects, and intervention durations. Even with a moderate probability of change in CD4 count (mean 5% change in CD4 category) for a small number patients (low utilization office-based deployment scenario), the computer-delivered intervention was cost-effective as judged by the conventional benchmark of $50,000 per QALY (cost per QALY = $32,829; Table 6). With higher probabilities of effects and wider deployment via the Internet, use of the intervention would result in net cost savings.

These results are similar to those of other analyses that have reported net cost savings from interventions to enhance adherence. Sansom et al. [19] showed that intensive case management for adherence produced net cost savings from decreased service utilization, although this study did not take increased medication costs into account. Our conclusions are also consistent with those of Goldie et al. [52] who argued that even adherence interventions with modest effects may be cost effective, and Freedberg et al. [53] who showed that a nurse-led adherence intervention was cost-effective with a cost per QALY of $14,100 compared to usual care. Further, Braithwaite et al. [31] showed via a sophisticated computer model of the effects of adherence on health status and costs in persons treated for HIV that a modestly effective intervention associated with an absolute risk reduction of 4% for poor adherence costing less than $1,000 per user would be cost effective (Table 6). Analyses presented here show that even with conservative projections of intervention effectiveness and with small-scale deployment, cost per user estimates for the intervention was less than $500 and in many instances was associated with net costs savings (Table 6).

Results thus emphasize the importance of the duration of the intervention’s effect in determining its cost effectiveness. In these analyses, a range of effect durations was used in sensitivity analyses as we lacked specific information on how long the effects of the intervention would persist. At very short durations of effectiveness, the cost per QALY of the intervention was unacceptably high, but with greater durations of effect cost savings and improved quality of life accrue, yielding a more favorable cost per QALY (less than $50,000). For analyses presented in Tables 6 and 7, a six month duration was assumed based on other data showing that the duration of effect of education-oriented adherence interventions may range up to eighteen months [50,51]. Further, analyses in a meta-analysis of trials of adherence interventions showed no marked deterioration in intervention effects over time after adherence interventions [27]. These findings highlight the need for better data on the impact of adherence interventions on actual adherence over time. As noted in a review of adherence interventions for HIV [54], many studies of adherence interventions do not include follow-up data. Given the relation of cost-effectiveness to duration of intervention effect found in our current analyses, it is clear that more information is needed on how long an intervention may be effective and on strategies to promote adherence over time.

Limitations of this study should be acknowledged. As with any cost-effectiveness or cost-utility analysis, it was necessary to make several assumptions about the effects of the intervention on patient medication adherence and on the effects of adherence on patient health. To address these issues, we based analyses on other researchers’ models of the relation of adherence to viral load and CD4 count [31], used conservative estimates of the health utilities associated with various levels of CD4 count [29], and used sensitivity analyses taking into account a range of possible levels of intervention effectiveness. We depended on the convention of judging the cost effectiveness of an intervention based on the criterion of more or less than $50,000 per QALY although we note that other investigators have argued for a range of values from $50,000 to $100,000 or more [48,49].

Others have shown that computer-delivered interventions may be cost-effective in health care. McCrone et al. [55], for example, showed that computer-delivered cognitive behavioral therapy could be a cost-effective strategy to deliver depression treatment in primary care, and Smith et al. [56] showed that a computer-delivered intervention for smoking cessation was also cost effective. The results presented here are also consistent with other studies that have shown that in spite of their development and deployment costs, computer-delivered interventions can provide good value in terms of health outcomes in relation to costs. This may be particularly relevant in delivery of services to persons in rural areas; other researchers have shown that telephone-delivered interventions may be effective in this population [57,58]. Analyses that included increased costs of transportation for rural patients to travel to a clinician’s office to use the intervention yielded cost per QALY estimated that were only slightly different from those found when these costs were not included. Analyses thus show that method and scope of deployment of an intervention can substantially affect its cost effectiveness, as the Internet-based deployment condition, with a large number of users, resulted in the greatest net cost savings. An additional limitation is that the results presented here assume that the effects of the intervention persist for a longer period than actually demonstrated in the original study. Although we note that other similar adherence interventions may have effects that persist for as long as one year [27], it should be acknowledged that this assumption is not directly supported by our data. Further, it should be noted that some of our cost estimates, notably the cost of office space, may vary widely by geographic locale.

Another important issue is the extent to which the effects of any intervention for adherence delivered over the Internet would mirror those obtained from the office-based information kiosk format. Although the content and types of interactions would be virtually identical, the context in which the intervention is completed may have an unknown impact on outcomes. Further, while patients may be more likely to complete this type of intervention when it is recommended in the clinician’s office, they may be less likely to spontaneously use it when it is provided over the Internet. It is noted that searching for health information is a common use of the Internet even among mobile phone users [59] and that in a recent article the director of the National Institutes of Health, Francis Collins, cited the development of health applications as a research priority [60]. Additional information on patients’ use of Internet-based health applications is thus needed.

Still another issue is related to the demands the intervention makes on motivation and attention on the part of affected patients, some of whom may be affected by mental health and substance abuse problems as well as economic and housing instability. During the development of the intervention, a large number of our participants were affected by these issues and still were able to complete not only the intervention but also a demanding battery of cognitive and psychosocial measures. As noted in the introduction, the intervention addressed the possible problems that might have been encountered by extensively testing the intervention prior to deployment with potential users.

Given the potential advantages of computer-delivered interventions, it is reasonable to question why they are not more widely used. Shakeshaft and Frankish [61] also note the possible advantages of computer-delivered interventions in prevention, and suggest several factors that may affect their adoption. They indicate that a combination of lack of understanding of existing evidence for effectiveness, perceived high cost of implementing the intervention, and concerns about the impact of less personal interventions may reduce health care providers’ enthusiasm for computer-delivered interventions. In our own clinic, we have encountered concerns about lack of space, the potential for lengthening patient visits thereby decreasing clinic efficiency, and the initial cost of computers in discussions of deploying computer-based interventions. It is thus likely that even with demonstration of cost effectiveness, deployment of the intervention as an information kiosk may face resistance.

While deployment of the intervention on the Internet may address factors related to the clinic, it will have its own drawbacks. These would include determining the most effective ways to ensure that patients become aware of the intervention, encouraging them to complete it, and the possibility that the patients most in need of the intervention may not have high-speed access to it on the Internet. The intervention’s multimedia and interactive format make it difficult to deploy on standard cellular telephones, most of which lack the types of screens and processors that could render the material on the telephone screen. Although the intervention can be viewed on a smartphone, only the increasing penetration of high speed data access and smart phones will allow the intervention to be easily accessed by persons without Internet-connected computers.

## Conclusions

These analyses show that in spite of higher initial development costs than for clinician-delivered interventions, a computer-delivered intervention may be a cost effective strategy for improving medication adherence in persons treated for HIV. The analyses also show that depending on the effectiveness of the intervention and on how widely it is deployed, its use may result in net cost savings. Each possible delivery format for adherence education has potential benefits. As summarized in Table 8, clinician-delivered interventions may be the most flexible and individually-tailored but may suffer from the time press of daily clinical practice. Electronic delivery of adherence interventions may still be tailored but less flexibly so. Electronic delivery may provide more detailed information in a multimedia format, but provide patients with less opportunity to ask questions. Electronic delivery formats raise the possibility that clinicians will actually spend less time on adherence with patients, although some evidence suggests that patients provided with access to electronically-delivered information may be more active participants in medical encounters [18]. Results of this study may be useful in supporting the development and deployment of other computer-delivered interventions. In light of the great need for better interventions to improve medication adherence and the lack of trained personnel to deliver them, a computer-delivered intervention may be helpful in addressing need while making small demands on treatment resources.

**Table 8:** Summary of advantages and disadvantages of intervention formats

**Delivery format**	**Advantages**	**Disadvantages**
**Clinician**	• Personal	• Time required
	• Information readily tailored to patient concerns or characteristics	• Oral presentation related to limited learning
		• Clinicians may not have therapeutic skills
	• Interactive	
	• Opportunity for questions and follow-up	
**Information Kiosk**	• Information can be computer tailored	• Less personal
	• Interactive	• Less clearly tailored
	• Multimedia may enhance patient interest and understanding	• Less opportunity for follow-up
	• Requires less clinician time	• Demands some computer skills
	• Can provide more information in a longer intervention	• Requires space and computer support
	• Can provide therapeutic interventions for depression and substance abuse	
**Internet Based**	• Information can be computer tailored	• Less personal
		• Less clearly tailored
		• Much less opportunity for follow-up questions
	• Interactive	
		• Requires computer and high-speed Internet access
	• Multimedia may enhance patient interest and understanding	
	• Requires less clinician time	
	• Can provide more information in a longer intervention	
	• Can provide therapeutic interventions for depression and substance abuse	
	• Can be available on demand at times when patients are available or motivated.	

## Competing interests

The authors declare that they have no competing interests.

## Authors’ contributions

RO designed the original study, obtained grant funding for its execution, completed data analyses, and wrote a draft of the original manuscript. DW collaborated in the design of the original study, assisted in interpretation of data analyses, and assisted in the preparation of the manuscript. RJ assisted in the development of the study intervention, assisted in completing the study, and helped to draft the manuscript. AA provided assistance in data analyses and helped to draft the manuscript. JC assisted in the development of the study intervention, assisted in completing the study, and helped to draft the manuscript. All authors read and approved the final manuscript.

## Pre-publication history

The pre-publication history for this paper can be accessed here:

http://www.biomedcentral.com/1472-6947/13/29/prepub

## Supplementary Material

Additional file 1 Screens from the current intervention. Click here for file

Additional file 2 Cost per QALY for Unadjusted Costs, Deployment Scenarios, Durations and Levels of Effectiveness (all values in US dollars). Click here for file
