# Supplementary material for: Cost effectiveness of a computer-delivered intervention to improve HIV medication adherence
Source: BMC Med Inform Decis Mak. 2013 Feb 28;13:29. doi: 10.1186/1472-6947-13-29 (PMC3599639; doi:10.1186/1472-6947-13-29)

APPENDIX. SCREENS FROM THE CURRENT INTERVENTION

Note: All material is provided via audio narration as well as via on screen text and graphics.

An initial screen includes a brief video that explains how to interact with the touch screen:

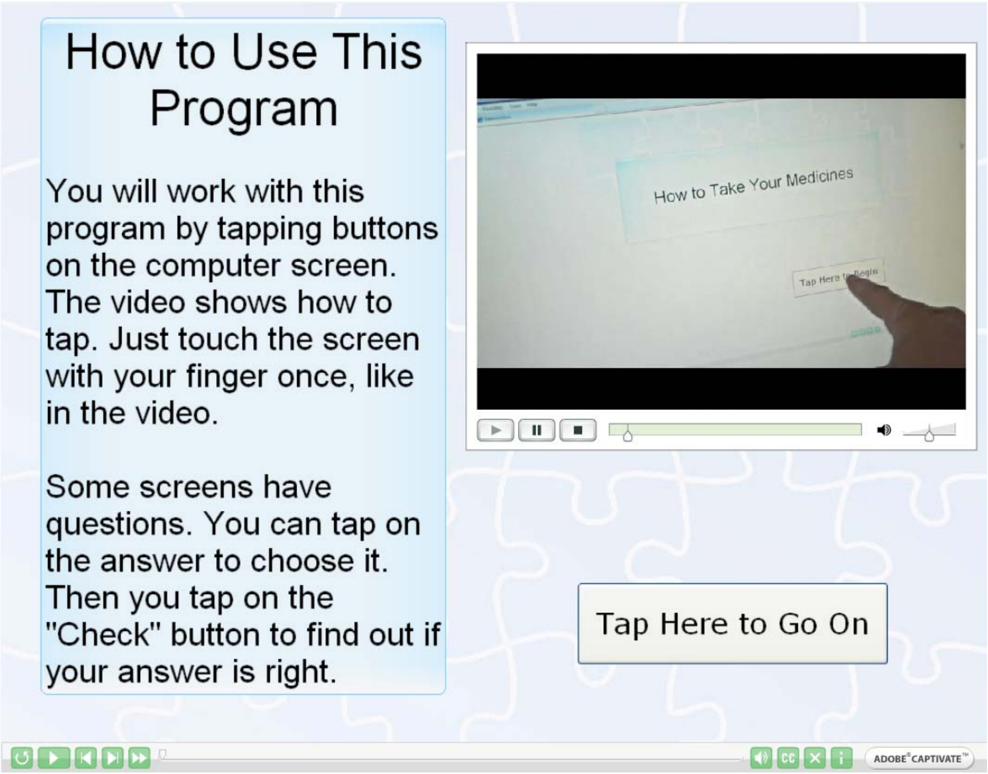

Participants enter their name or nickname to provide personalization:

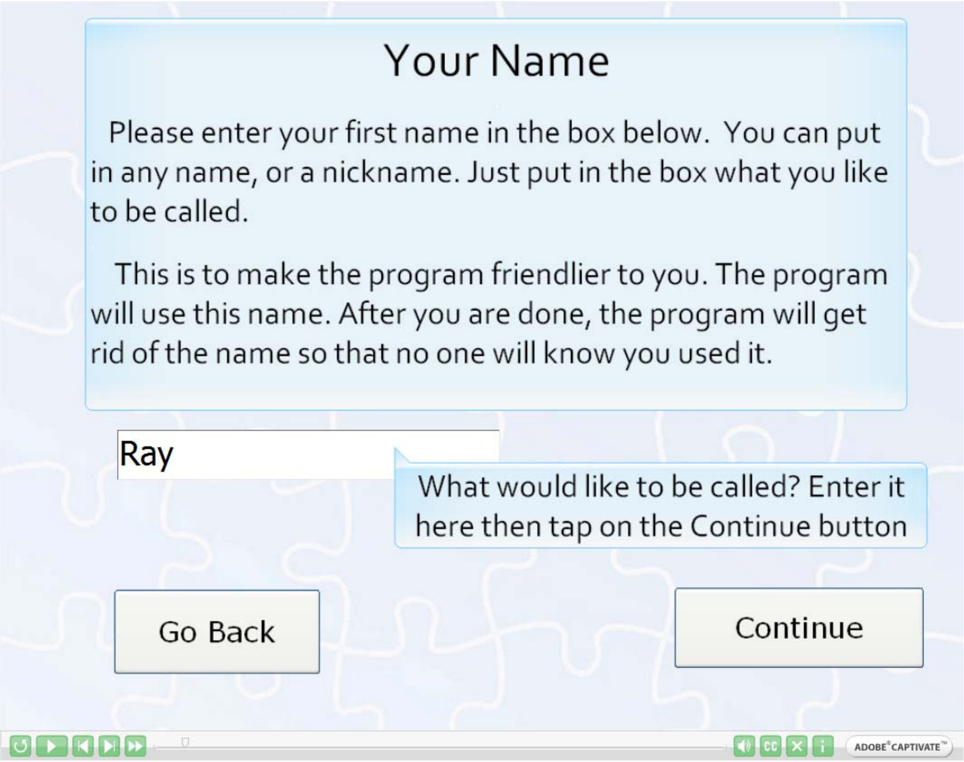

The program includes an early self-check question to ensure that the participant understands how to respond to questions. This picture also illustrates the program's response using the participant's name:

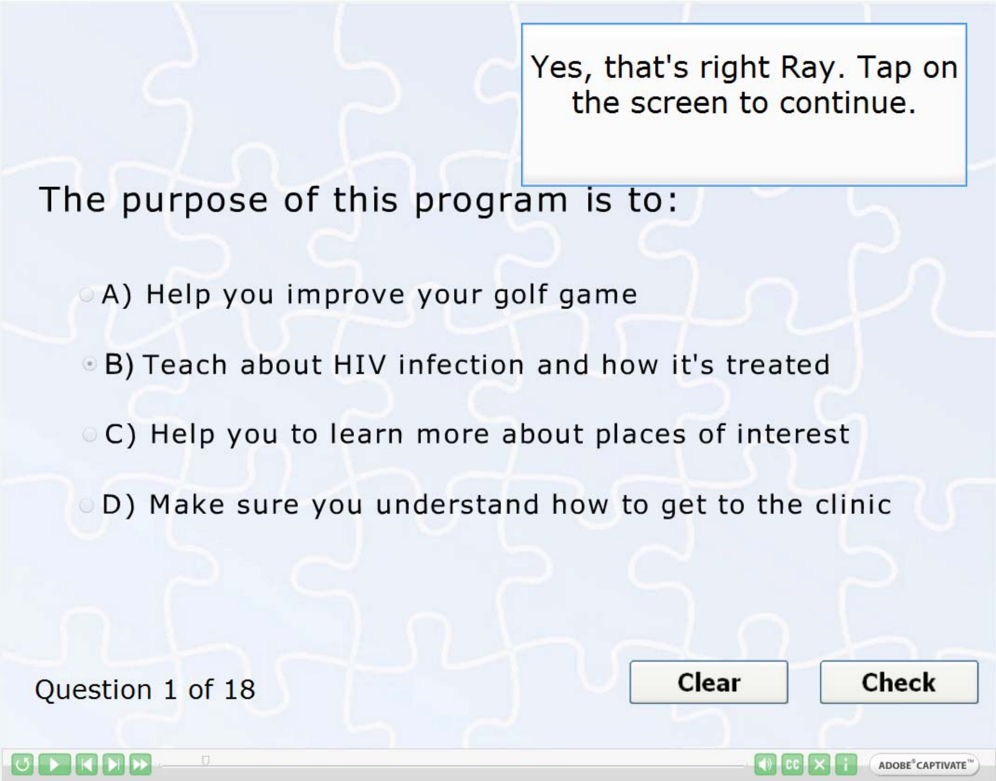

The program then provides an initial overview of its content:

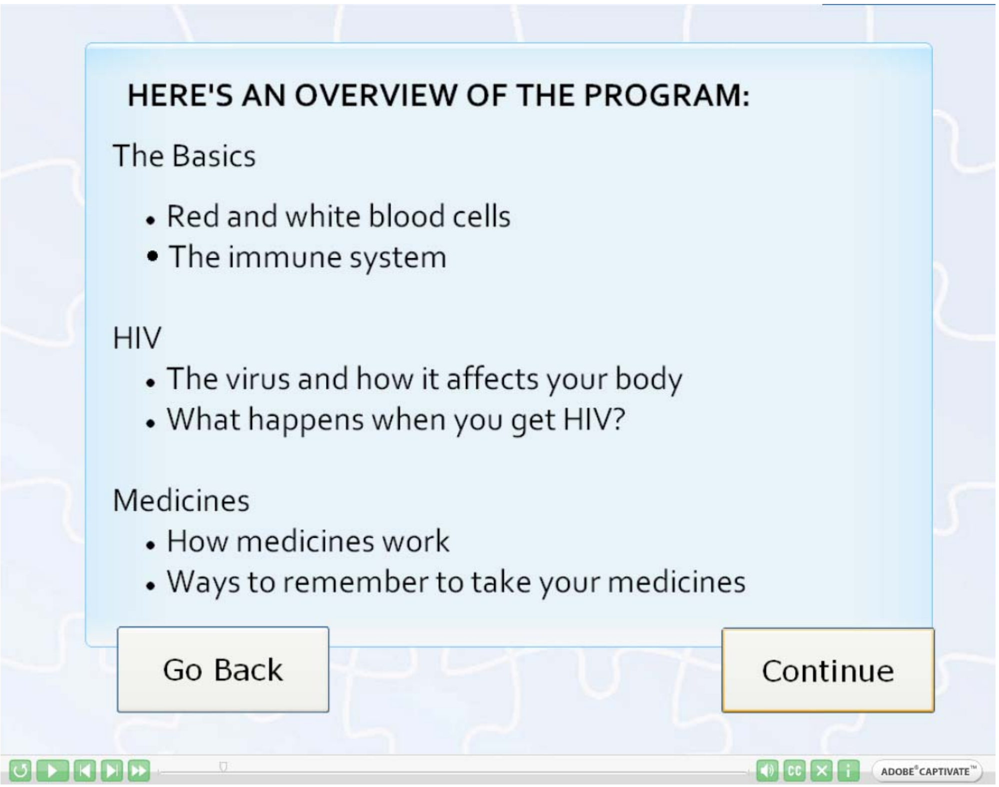

The intervention includes an explanation of what CD4 or T cells are and the existence of receptors:

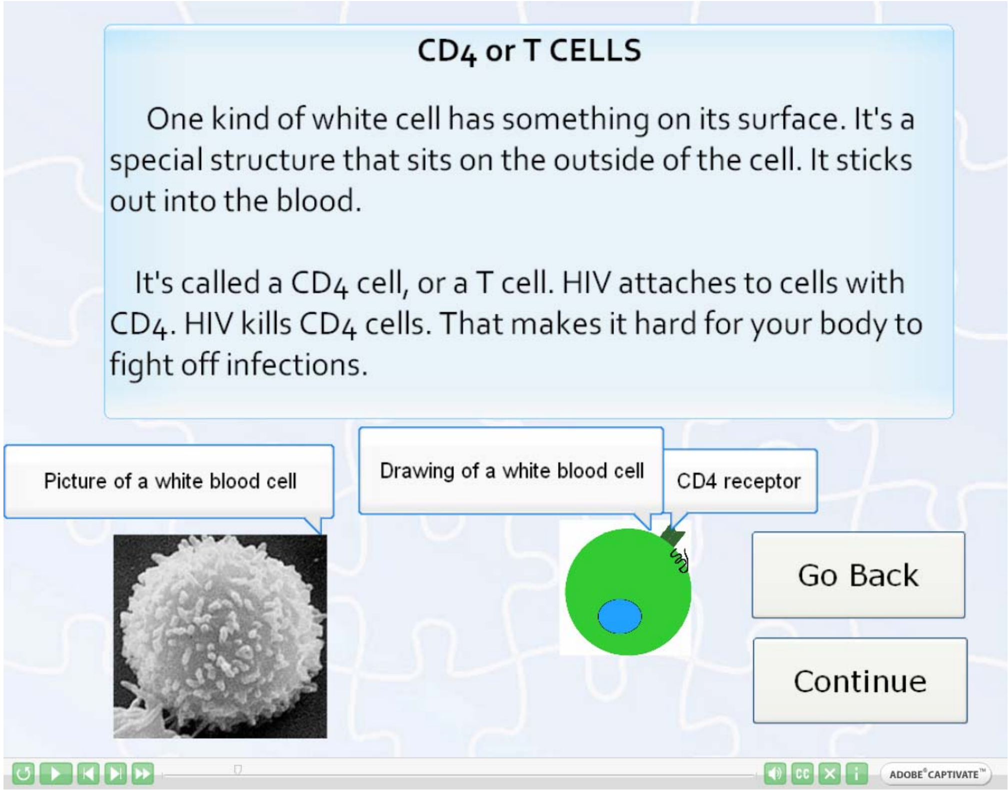

An animation provides an overview of the viral life cycle before the program goes through it again, step by step:

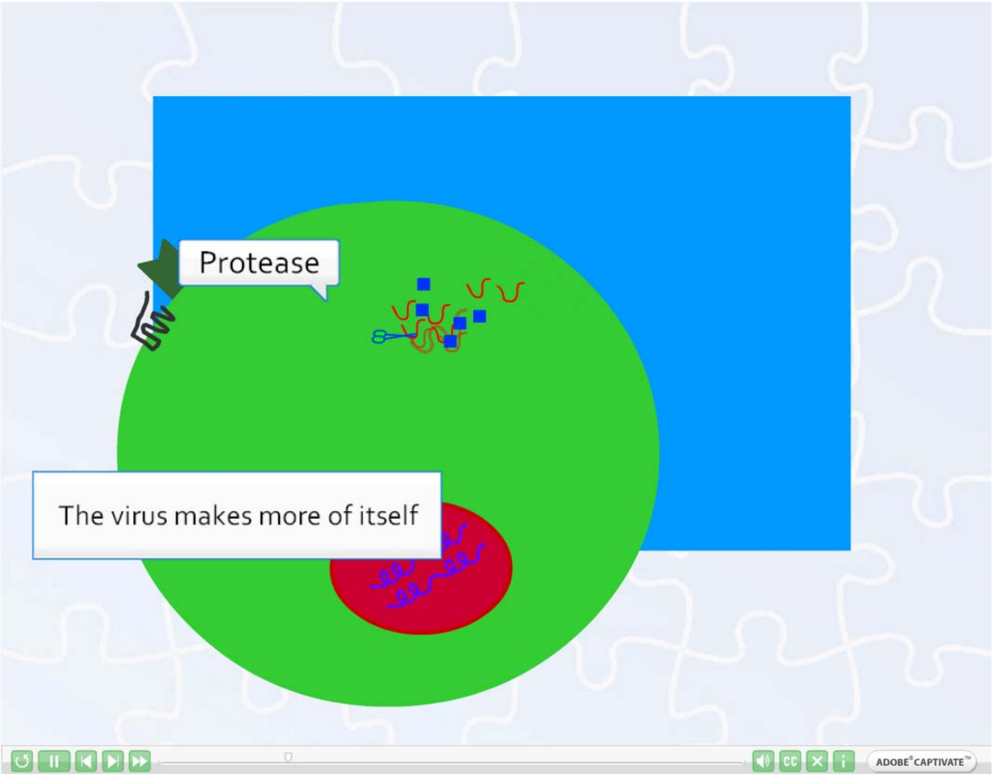

A second review of the viral life cycle emphasizes mechanisms of drug action:

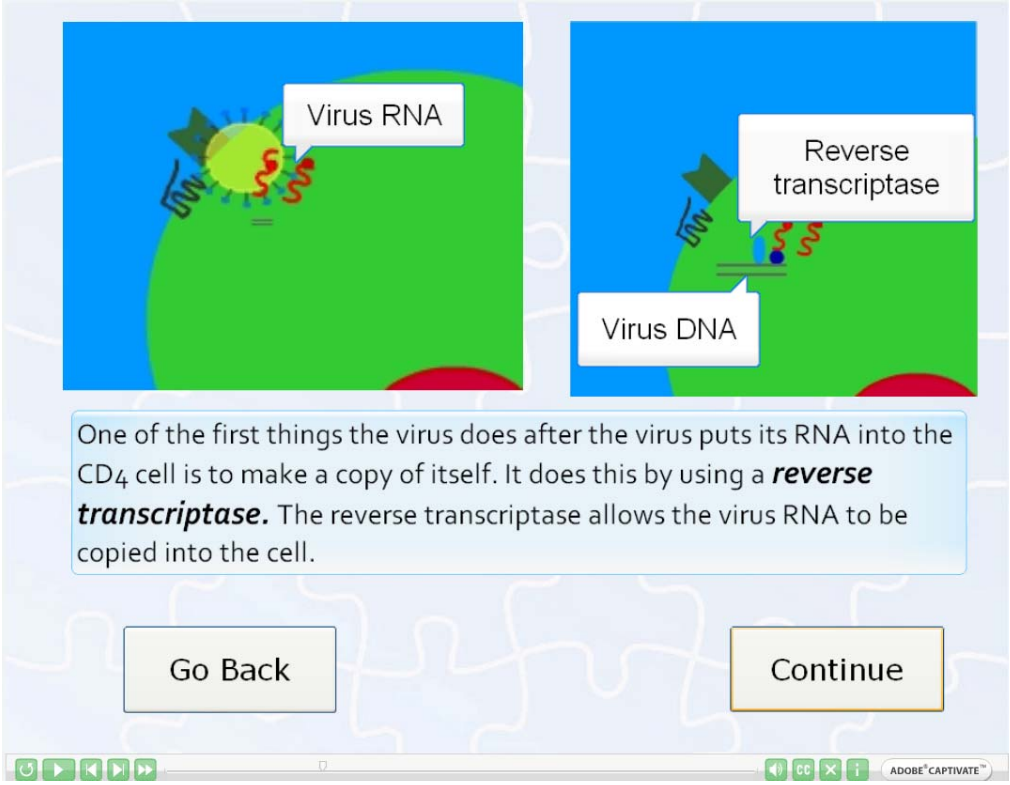

Self check questions help the participant determine whether they understand content:

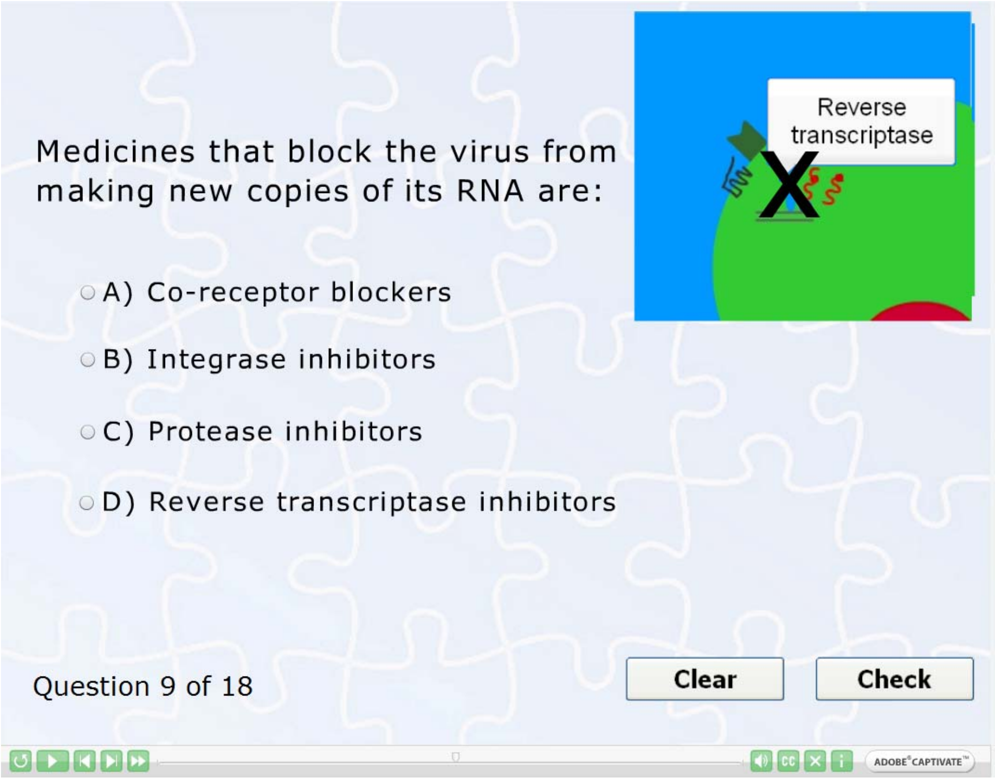

If persons do not answer correctly, the program lets them know and then provides a review:

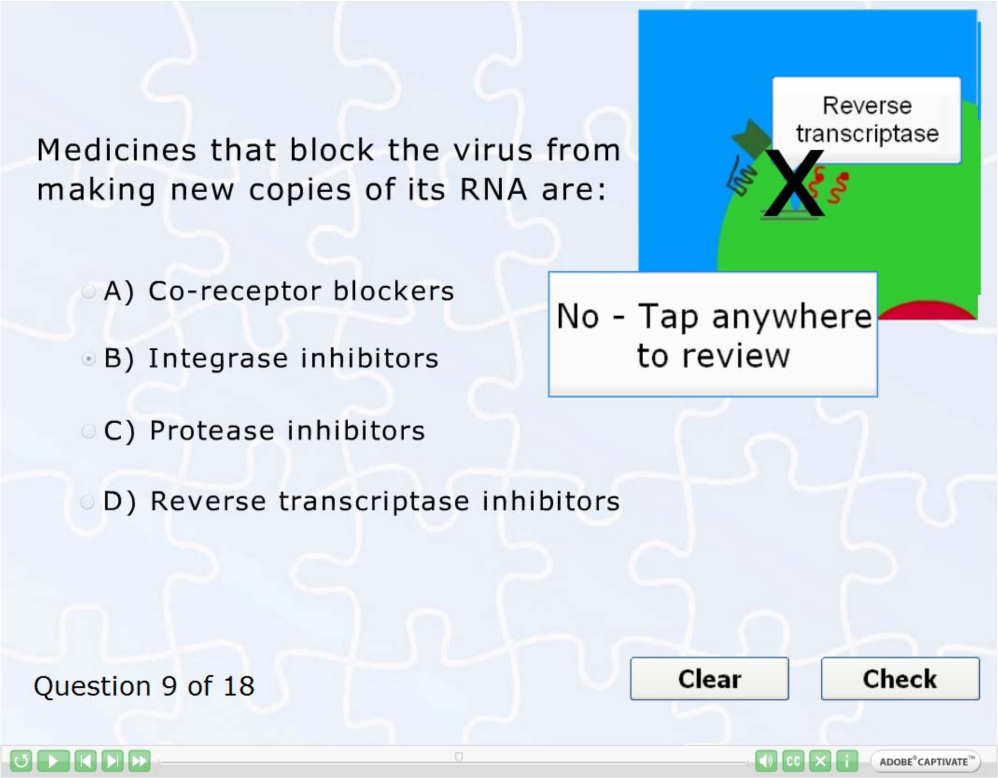

Review material emphasizes a single key concept. Persons who answer the question correctly do not see the review material.

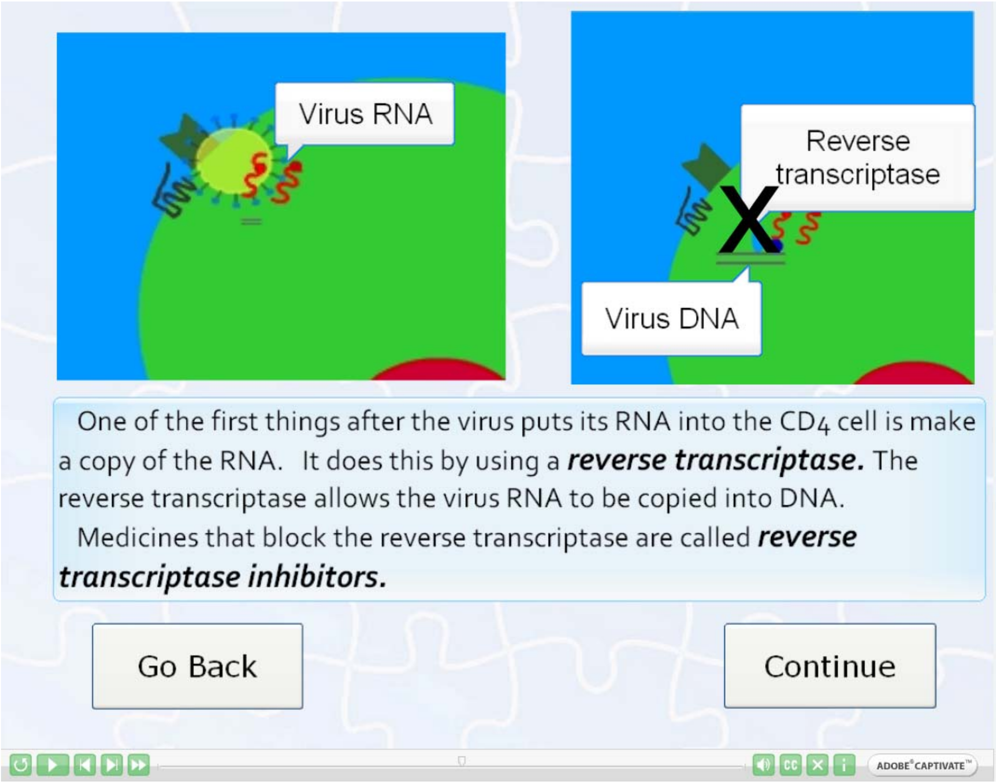

The program includes material on coping with various barriers to adherence, including a brief discussion of altering self talk to cope with stigmatizing attitudes of others:

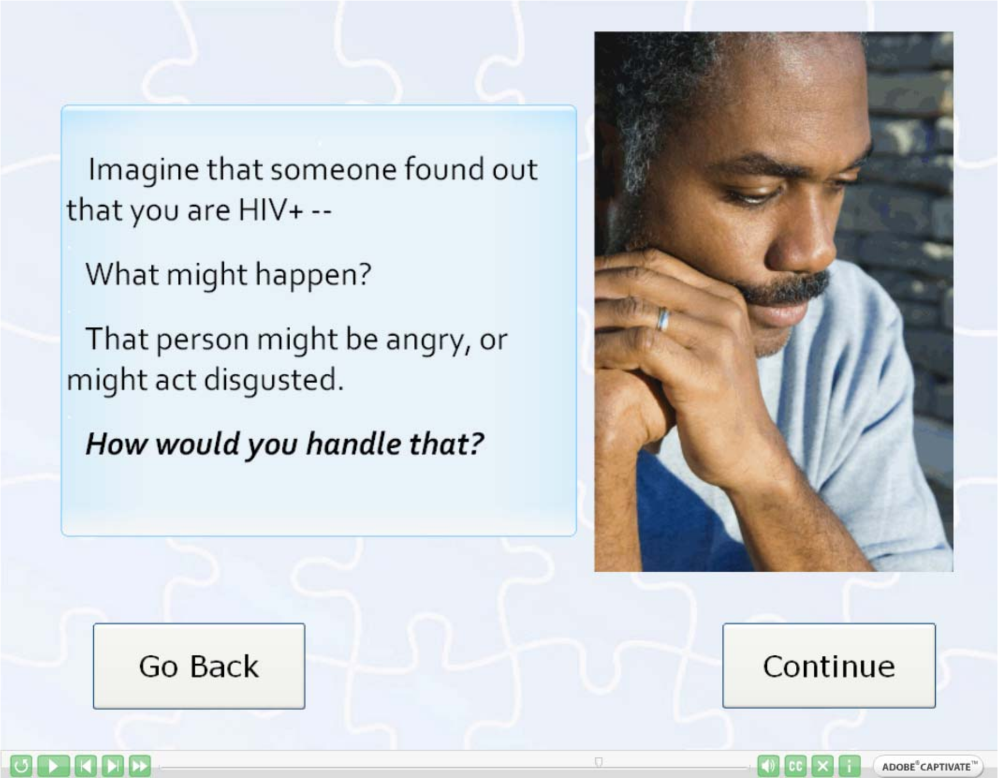

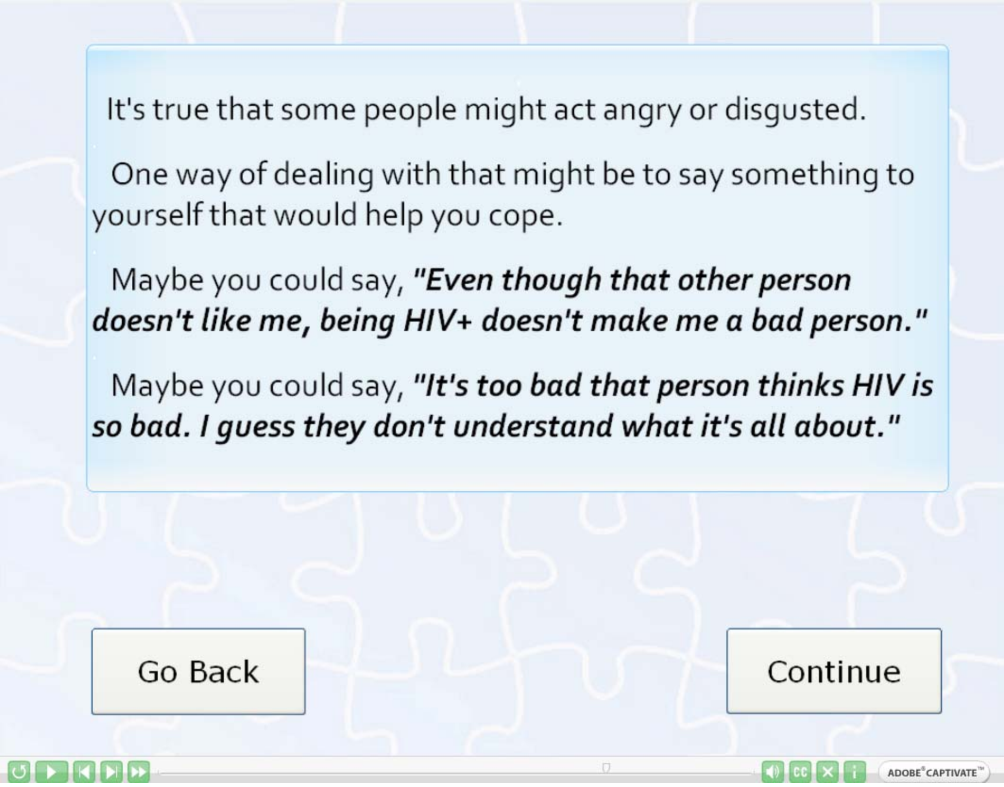

Other content explains in concrete terms the need for and meaning of viral load testing and immune status markers. Please note that this is only a brief extract of a much longer step-by-step explanation:

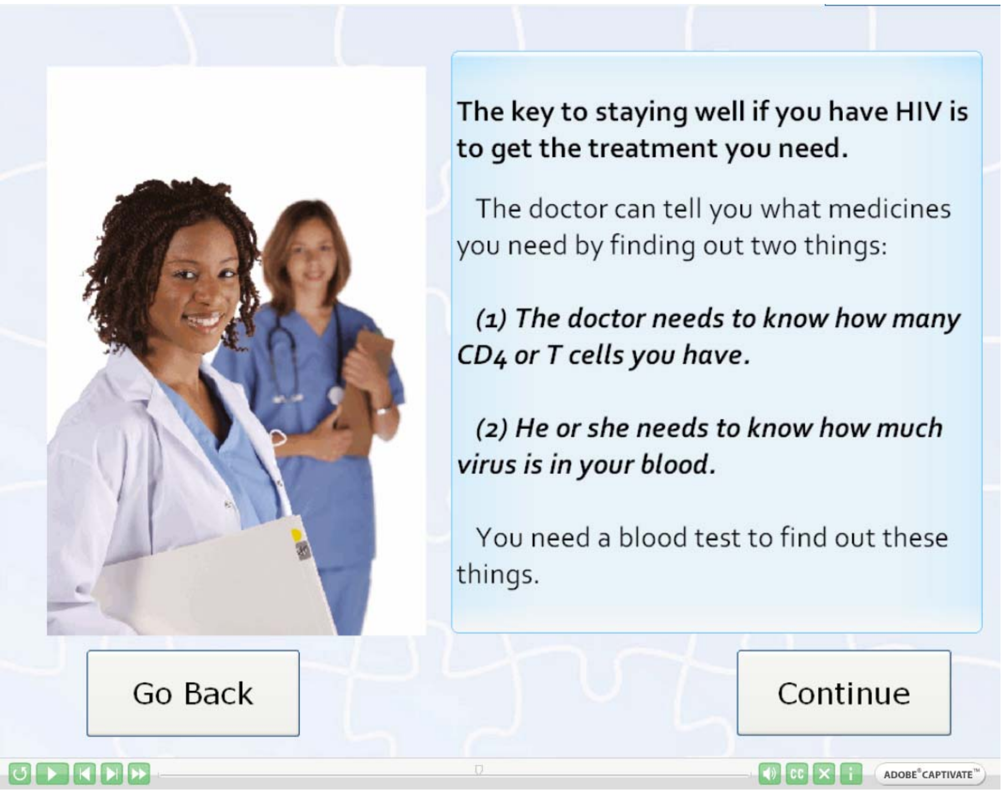

The program also includes a graphic explanation of the meaning of high levels of adherence based on research on communicating quantitative concepts to patients:

You have to be very good at taking your medicine if you want to stay healthy.

Studies show that people who take their medicine almost all of the time are more likely to have undetectable viral loads.

When the viral load is very low, most people feel good and can get on with their lives.

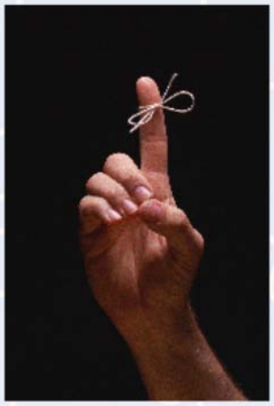

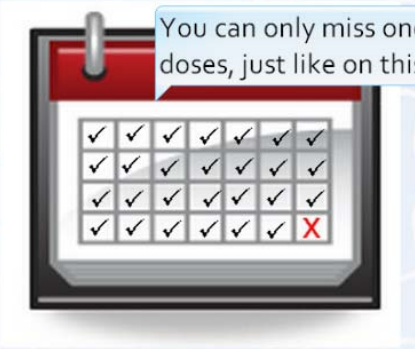

You can only miss one or two doses, just like on this calendar.

Go Back

Continue

The program includes a series of screens on coping with side effects and common misconceptions, such as not taking antiretrovirals if one has a drink of alcohol:

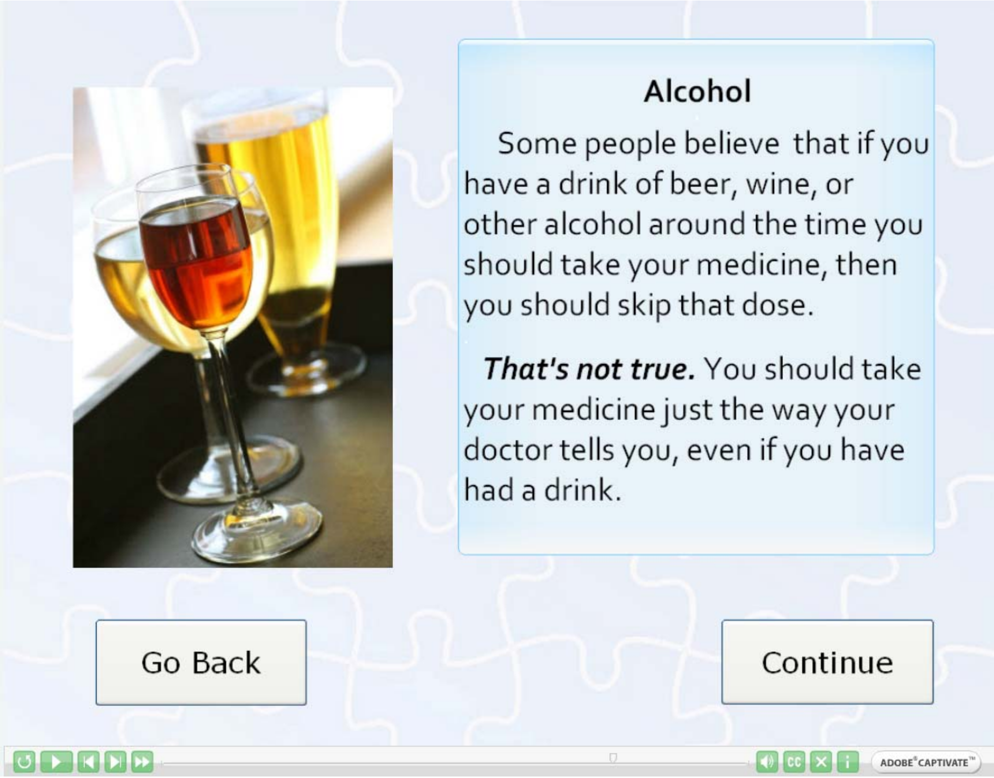

A final module reviews strategies for adherence, such as using a pillbox:

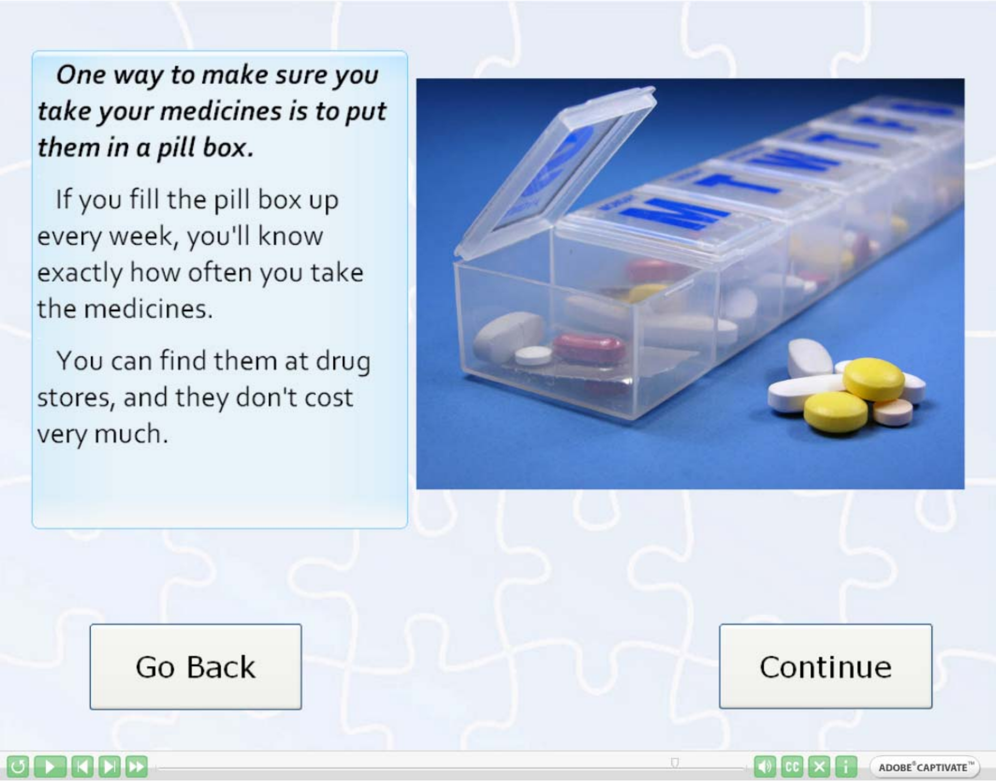

The adherence module concludes by asking the participant to state whether they have a strategy for adherence and thus elicits a commitment from them on adherence:

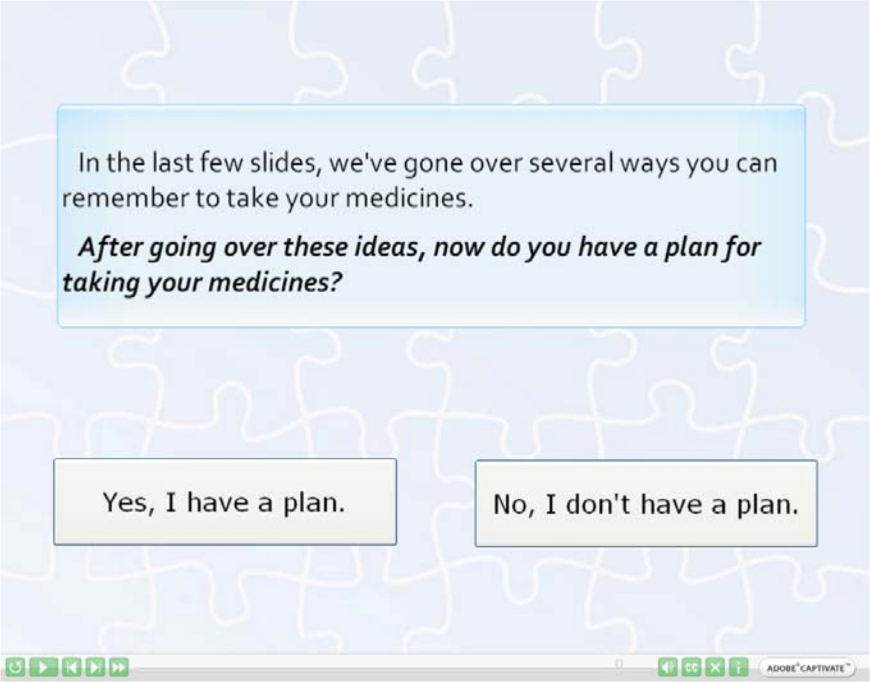

If participants indicate that they have a plan, a response screen provides praise and reinforces the importance of high levels of adherence. If the participant indicates that he or she does not have a plan, the response screen informs the participant that having a plan is a good way to ensure high levels of adherence and offers to review the material on adherence once again.

Based on feedback from consumers during usability testing and our experience in an earlier study, the program provides participants the opportunity to print out material to take home:

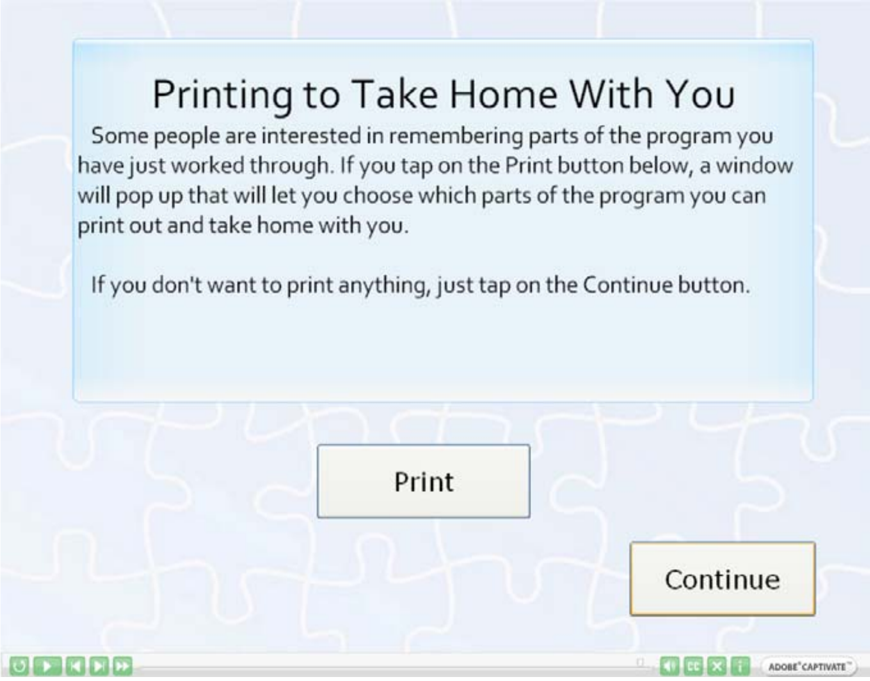

Supplement: Additional file 1 — Screens from the current intervention. [file 1472-6947-13-29-S1.pdf]
