# Supplementary material for: Cost effectiveness of a computer-delivered intervention to improve HIV medication adherence
Source: BMC Med Inform Decis Mak. 2013 Feb 28;13:29. doi: 10.1186/1472-6947-13-29 (PMC3599639; doi:10.1186/1472-6947-13-29)
Supplement: Additional file 2 — Cost per QALY for Unadjusted Costs, Deployment Scenarios, Durations and Levels of Effectiveness (all values in US dollars). [file 1472-6947-13-29-S2.pdf]

Supplemental Tables
Cost per QALY for Unadjusted Costs, Deployment Scenarios, Durations and Levels of Effectiveness (all values in US dollars)

Note: *Duration* refers to the assumed amount of time the duration has an effect on patient status; *Effectiveness* refers to the average probability of patients making a transition from one CD4-related status to the next highest group; *Rural vs nonrural* indicates whether transportation costs were included in deployment cost calculations; *Unadjusted costs* indicates that the costs were not reduced for costs that could be specifically attributed to research expenses.

Cost Multiplier	Low Office	High Office	Low Web	High Web
Cost/QALY with **1** month duration @ **1%** effectiveness, nonrural, and unadjusted costs				
-0.5	1,876,967	658,929	166,382	-40,253
-0.25	2,790,495	963,438	318,637	10,449
0.0	3,704,024	1,267,948	470,892	61,250
0.25	4,617,552	1,572,457	623,146	112,002
0.5	5,531,081	1,876,967	775,401	162,754
Cost/QALY with **3** month duration @ **1%** effectiveness, nonrural, and unadjusted costs				
-0.5	201,850	66,512	56,797	-14,501
-.25	303,353	100,347	107,548	2,416
0.0	404,856	134,181	158,300	19,334
0.25	506,359	168,016	209,051	36,251
0.5	607,863	201,850	259,803	53,168
Cost/QALY with **6** month duration @ **1%** effectiveness, nonrural, and unadjusted costs				
-0.5	39,805	5,970	29,400	-8063
-0.25	65,181	14,429	54,776	396
0.0	90,556	22,888	80,152	8,854
0.25	115,932	31,346	105,528	17,313
0.5	141,308	39,805	130,903	25,772
Cost/QALY with **12** month duration @ **1%** effectiveness, nonrural, and unadjusted costs				
-0.5	-2,708	-11,166	15,702	-4,844
-0.25	3,636	-9052	28,390	-614
0.0	9,980	-6,937	41,078	3,615
0.25	16,324	-4,822	53,766	7,844
0.5	22,668	-2,708	66,454	12,073

Cost/QALY with **1** month duration @ **2%** effectiveness, nonrural, and unadjusted costs				
Delta cost	Office small	Office large	Online small	Online large
-0.5	1,875,074	657,036	155,960	-50,945
-0.25	2,788,603	961,546	607,944	-194
0.0	3,702,131	1,266,055	460,199	50,558
.25	4,615,660	1,570,565	612,454	101,309
.5	5,529,188	1,875,074	764,709	152,061
Cost/QALY with **3** month duration @ **2%** effectiveness, nonrural, and unadjusted costs				
-0.5	199,958	64,620	52,504	-18,793
-0.25	301,461	98,454	103,256	-1,876
0.0	402,964	132,289	154,007	15,041
0.25	504,467	166,123	204,759	31,958
0.5	605,970	199,958	255,511	48,876
Cost/QALY with **6** month duration @ **2%** effectiveness, nonrural, and unadjusted costs				
-0.5	37,913	4,078	26,708	-10,755
-0.25	63,288	12,537	52,084	-2,296
0.0	88,664	20,995	77,459	6,162
0.25	114,040	29,454	102,835	14,621
0.5	139,416	37,913	128,211	23,079
Cost/QALY with **12** month duration @ **2%** effectiveness, nonrural, and unadjusted costs				
-0.5	-4,600	-13,058	13,810	-6,736
-0.25	1,744	-10,944	26,498	-2,507
0.0	8,088	-8,829	39,185	1,723
0.25	14,432	-6,715	51,873	5,952
0.5	20,776	-4,600	64,561	10,181
Cost/QALY with **1** month duration @ **5%** effectiveness, nonrural, and unadjusted costs				
Delta cost	Office small	Office large	Online small	Online large
-0.5	1,842,327	624,289	-69,072	-275,707
-0.25	2,755,855	928,798	83,182	-224,956
0.0	3,669,384	1,233,308	235,437	-174,204
0.25	4,582,912	1,537,817	387,692	-123,452
0.5	5,496,441	1,842,327	539,947	-72,701

Cost/QALY with **3** month duration @ **5%** effectiveness, nonrural, and unadjusted costs				
-0.5	167,210	31,873	-32,611	-103,908
-0.25	268,713	65,707	18,141	-86,991
0.0	370,213	99,541	68,892	-70,074
0.25	471,720	133,376	119,644	-53,157
0.5	573,223	167,210	170,395	-36,239

Cost/QALY with **6** month duration @ **5%** effectiveness, nonrural, and unadjusted costs				
-0.5	5,165	-28,669	-23,496	-60,958
-0.25	30,541	-20,211	1,880	-52,500
0.0	55,917	-11,752	27,256	-44,041
0.25	81,292	-3,294	52,632	-35,583
0.50	106,668	5,165	78,008	-27,124

Cost/QALY with **12** month duration @ **5%** effectiveness, nonrural, and unadjusted costs				
-0.5	-37,347	-45,806	-18,938	-39,484
-0.25	31,003	-43,691	-6,250	-35,254
0.0	-24,659	-41,577	6,438	-31,025
0.25	-18,316	-39,462	19,126	-26,796
0.50	-11,972	-37,347	31,814	-22,566

Cost/QALY with **1** month duration @ **9%** effectiveness, nonrural, and unadjusted costs				
-0.5	1,793,772	575,734	-471,505	-678,139
-0.25	2,707,301	880,244	-319,250	-627,388
0.0	3,620,829	1,184,753	-166,995	-576,636
0.25	4,534,358	1,489,263	-14,740	-525,885
0.5	5,447,886	1,793,772	137,514	-475,133

Cost/QALY with **3** month duration @ **9%** effectiveness, nonrural, and unadjusted costs				
-0.5	118,656	-16,682	-177,678	-248,975
-0.25	220,159	17,152	-126,926	-232,058
0.0	321,662	50,987	-76,174	-215,141
0.25	423,165	84,821	-25,423	-198,223
0.5	524,668	118,656	25,329	-181,306

Cost/QALY with **6** month duration @ **9%** effectiveness, nonrural, and unadjusted costs				
-0.5	-43,390	-77,224	-104,221	-141,684

-0.25	-18,014	-68,765	-78,845	-133,225
0.00	7,362	-60,307	-53,469	-124,767
0.25	32,738	-51,848	-28,093	-116,308
0.50	58,114	-43,390	-2,718	-107,849
Cost/QALY with **12** month duration @ **9%** effectiveness, nonrural, and unadjusted costs				
-0.5	-85,902	-94,361	-67,492	-88,038
-0.25	-79,558	-92,246	-54,805	-83,809
0.00	-73,214	-90,131	-42,117	-79,580
0.25	-66,870	-88,017	-29,429	-75,350
0.50	-60,526	-85,902	-16,741	-71,121
Cost/QALY with **1** month duration @ **5%** effectiveness, with **rural** transport, unadjusted costs				
-.5	1,866,863	648,825	N/A	N/A
-0.25	2,780,392	953,335	N/A	N/A
0.00	3,693,920	1,257,844	N/A	N/A
0.25	4,607,449	1,562,354	N/A	N/A
0.50	5,520,977	1,866,863	N/A	N/A
Cost/QALY with **3** month duration @ **5%** effectiveness, with **rural** transport, unadjusted costs				
-.50	175,389	40,051	N/A	N/A
-0.25	276,892	73,886	N/A	N/A
0.00	378,395	107,720	N/A	N/A
0.25	479,898	141,555	N/A	N/A
0.50	581,402	175,389	N/A	N/A
Cost/QALY with **6** month duration @ **5%** effectiveness, with **rural** transport, unadjusted costs				
-.50	9,254	-24,580	N/A	N/A
-.25	34,630	-16,121	N/A	N/A
0.00	60,006	-7,663	N/A	N/A
0.25	85,382	796	N/A	N/A
0.50	110,758	9,254	N/A	N/A
Cost/QALY with **12** month duration @ **5%** effectiveness, with **rural** transport, unadjusted costs				
-.50	-35,303	-43,761	N/A	N/A
-.25	-28,959	-41,647	N/A	N/A
0.00	-22,615	-39,532	N/A	N/A
0.25	-16,271	-37,417	N/A	N/A

0.50	-9,927	-35,303	N/A	N/A
